# Systematic Review and Meta‐Analysis of Prehospital Machine Learning Scores as Screening Tools for Early Detection of Large Vessel Occlusion in Patients With Suspected Stroke

**DOI:** 10.1161/JAHA.123.033298

**Published:** 2024-06-11

**Authors:** Muath Alobaida, Martha Joddrell, Yalin Zheng, Gregory Y. H. Lip, Fiona J. Rowe, Wahbi K. El‐Bouri, Andrew Hill, Deirdre A. Lane, Stephanie L. Harrison

**Affiliations:** ^1^ Liverpool Centre for Cardiovascular Science University of Liverpool, Liverpool John Moores University and Liverpool Heart & Chest Hospital Liverpool UK; ^2^ Department of Cardiovascular and Metabolic Medicine Institute of Life Course and Medical Sciences, University of Liverpool Liverpool UK; ^3^ Department of Basic Science, Prince Sultan Bin Abdulaziz College for Emergency Medical Services King Saud University Riyadh Saudi Arabia; ^4^ Department of Eye and Vision Sciences Institute of Life Course and Medical Sciences, University of Liverpool Liverpool UK; ^5^ Danish Centre for Health Services Research, Department of Clinical Medicine Aalborg University Aalborg Denmark; ^6^ Institute of Population Health, University of Liverpool Liverpool UK; ^7^ Department of Medicine, Whiston Hospital, St Helens and Knowsley Teaching Hospitals NHS Trust Liverpool UK; ^8^ Registry of Senior Australians South Australian Health and Medical Research Institute Adelaide Australia

**Keywords:** artificial intelligence, endovascular thrombectomy, large vessel occlusion, machine learning, prehospital, stroke, Diagnostic Testing, Ischemic Stroke, Cerebrovascular Disease/Stroke, Machine Learning, Cerebrovascular Procedures

## Abstract

**Background:**

Enhanced detection of large vessel occlusion (LVO) through machine learning (ML) for acute ischemic stroke appears promising. This systematic review explored the capabilities of ML models compared with prehospital stroke scales for LVO prediction.

**Methods and Results:**

Six bibliographic databases were searched from inception until October 10, 2023. Meta‐analyses pooled the model performance using area under the curve (AUC), sensitivity, specificity, and summary receiver operating characteristic curve. Of 1544 studies screened, 8 retrospective studies were eligible, including 32 prehospital stroke scales and 21 ML models. Of the 9 prehospital scales meta‐analyzed, the Rapid Arterial Occlusion Evaluation had the highest pooled AUC (0.82 [95% CI, 0.79–0.84]). Support Vector Machine achieved the highest AUC of 9 ML models included (pooled AUC, 0.89 [95% CI, 0.88–0.89]). Six prehospital stroke scales and 10 ML models were eligible for summary receiver operating characteristic analysis. Pooled sensitivity and specificity for any prehospital stroke scale were 0.72 (95% CI, 0.68–0.75) and 0.77 (95% CI, 0.72–0.81), respectively; summary receiver operating characteristic curve AUC was 0.80 (95% CI, 0.76–0.83). Pooled sensitivity for any ML model for LVO was 0.73 (95% CI, 0.64–0.79), specificity was 0.85 (95% CI, 0.80–0.89), and summary receiver operating characteristic curve AUC was 0.87 (95% CI, 0.83–0.89).

**Conclusions:**

Both prehospital stroke scales and ML models demonstrated varying accuracies in predicting LVO. Despite ML potential for improved LVO detection in the prehospital setting, application remains limited by the absence of prospective external validation, limited sample sizes, and lack of real‐world performance data in a prehospital setting.

**Nonstandard Abbreviations and Acronyms**
LVO: large vessel occlusion
ML: machine learning
NIHSS: National Institutes of Health Stroke Scale
SROC: summary receiver operating characteristic

**Clinical Perspective**

**What Is New?**

- This systematic review and meta‐analysis compared the accuracies of prehospital stroke scales and machine learning models for detecting large vessel occlusion, offering novel insights into their performance.

**What Are the Clinical Implications?**

- There may be potential for improved detection of large vessel occlusion with machine learning models, but the potential benefits would need to be weighed against additional resource requirements to implement the models in clinical practice.
- Caution is advised in definitive recommendations due to existing heterogeneity, population variation, and lack of prehospital data.
- Emphasizing the need for prospective multicenter studies is crucial to validate the practicality of integrating machine learning into prehospital settings.

The management of acute ischemic stroke with large vessel occlusion (LVO) is time dependent, with a critical need for prompt intervention. The increased demand for ambulance services necessitates early identification of LVO, as it is essential for improving treatment accessibility, minimizing brain damage, achieving better clinical outcomes, and effectively allocating resources.^1^

Stroke is a heterogeneous condition and its diagnosis requires more neuroimaging. Therefore, prehospital detection of LVO is challenging, as there is no single clinical score that is highly sensitive and specific.^2^

Current prehospital stroke scales are primarily focused on the presence of neurological symptoms and are used to detect suspected stroke patients for further diagnostic tests. Many of these scales were derived from the National Institute of Health Stroke Scale (NIHSS), but each scale has its own limitations.^3^ The variation in location and severity of LVO occlusion can affect the patient's symptoms and detection, and some patients with LVO may have atypical symptoms or no symptoms at all. This can make it difficult to identify patients with LVO, especially in prehospital settings where time is critical. Hence, enhancing the performance of these scales and balancing their sensitivity and specificity are necessary to ensure the rapid identification and prompt treatment of patients with LVOs while minimizing overdiagnosis that would burden limited stroke facilities.^4^ It may be possible to improve the accuracy of prehospital stroke scales by transitioning from traditional linear models (between presence of clinical symptoms and presence of LVO) to machine learning (ML) models that can better capture the intricate relationships among risk factors. For example, ML models can be trained to consider the interactions between different clinical symptoms, in addition to age, sex, and medical history, for better prediction. K‐nearest neighbors, random forest (RF), artificial neural network, and extreme gradient boosting (XGB) are 4 supervised learning algorithms that can be used for classification and regression tasks. K‐nearest neighbors classifies new data points by finding the most similar data points in the training set. RF combines multiple decision trees and aggregates their predictions. Artificial neural network learns to recognize patterns in data through adjusting weights to minimize errors through model layer. XGB is a gradient boosting algorithm that builds an ensemble of models which correct the errors of the previous model.

This systematic review aims to compare the performance of ML models with prehospital stroke scales for LVO detection. The findings of this review will help to inform the early stages of ML symptoms‐analysis evidence.

## Methods

The data that support the findings of this study are available from the corresponding author upon reasonable request. This study did not involve experimental animals or human subjects research, and therefore institutional review board review is not required.

### Eligibility Criteria

Studies eligible for this review compared ML models and prehospital stroke scales for predicting LVO at the prehospital or triage stage of emergency departments. The included population were individuals with suspected ischemic stroke with LVO who were potential candidates for thrombectomy. Studies were retrospective or prospective observational cohort, or cross‐sectional studies and post hoc analyses. Commentaries or letters to editors, abstracts only, and preprints were excluded. The primary outcome of interest was LVO defined as acute occlusion of the internal carotid artery, middle cerebral artery, basilar artery, vertebral artery, and tandem occlusion.^5^ The secondary outcomes of interest were intracerebral hemorrhage and subarachnoid hemorrhage defined as defined as bleeding in brain parenchyma or bleeding in subarachnoid space, respectively.^6^ Studies were included where performance metrics such as area under the curves (AUC) were available to compare the prediction performance of outcomes.

Studies that used brain images as input data for LVO determination were excluded given that most prehospital care lacks computed tomography scans for stroke diagnosis and even mobile stroke units equipped with computed tomography scans are still not widely available. Further studies aiming to detect stroke mimics or using natural language processing were excluded on the basis of using text as input data that are not related to the research question.

### Search Strategies and Selection

Six databases, MEDLINE, PubMed, CINAHL, Web of Science, Scopus, and Embase, were systematically searched for relevant articles from inception to October 10, 2023, restricted to English language only. The search strategy is detailed in Table S1. The protocol of this review was registered in the International Prospective Register of Systematic Reviews as CRD42022352708 (https://www.crd.york.ac.uk/prospero/) and conducted in line with Preferred Reporting Items for Systematic Reviews and Meta‐Analyses for diagnostic test accuracy guidelines.^7^

### Study Screening

The searches were exported to the reference management library (Endnote X9) and duplicates were removed. Titles, abstracts, and full text were screened independently by 2 reviewer authors (M.A. and M.J.). Any disagreement regarding title and abstract or full‐text screening decisions were resolved by discussion in line with the eligibility criteria. Any discrepancies between reviewers regarding title and abstract or full‐text screening decisions was settled through discussion. Rayyan^8^ was used for the blind screening and selection of studies independently.

### Data Extraction

Two independent authors (M.A. and M.J.) extracted relevant data from the eligible studies. Data extraction criteria included author, study design, publication date, publication country, demographics including age, sex and country of origin, sample size (training, test, and validation cohorts), outcomes of interest, missing data, prehospital scales used, ML models used, variables included in both prehospital and ML models, ML hyperparameters, model performance, model validation, feature or variable importance, software used, and code availability. Authors were contacted to obtain missing information where required, twice over a 1‐month period.

### Data Synthesis

A narrative synthesis of the included studies was performed on the extracted data. Meta‐analyses of AUC were conducted using the MedCalc statistical software package (https://www.medcalc.org/) for studies comparing different prehospital stroke scales and different ML models on LVO ischemic stroke. Meta‐analyses were performed on the AUCs and 95% CIs to pool the model's performance, considering that studies used different prehospital scales and different ML models to evaluate the primary outcome, LVO, and the secondary outcomes of intracerebral hemorrhage and subarachnoid hemorrhage.

Studies that reported the AUC and 95% CI or SE of both models and scales were eligible for the meta‐analyses. Where these data were missing, authors were contacted via email twice to obtain this information, before being excluded from the meta‐analyses where data were not available. Also, the diagnostic accuracy data (true/false positives and true/false negatives) were calculated from the available sensitivities, specificities, positive predictive values and negative predictive values of the included studies. Then, the available sensitivity and specificity of prehospital stroke scales and ML models were pooled separately in forest plot. Also, a summary receiver operating characteristic (SROC) curve and the AUC values were generated using of the bivariate model with the package “midas” in STATA 14.

The *I* ^2^ and Cochran's Q statistic were used to assess heterogeneity between studies and visually through inspection of the AUC, forest plots, and SROC curves. Also, significant statistical heterogeneity was defined as *I* ^2^>50% and Q‐test *P*≤0.10.^9^ If the Q‐test or *I* ^2^ suggested significant heterogeneity, the AUCs were pooled using a random‐effects models. Otherwise, fixed‐effects models were used to pool AUCs and their CIs of the prehospital stroke scales and ML models.^10^

Despite logistic regression (LR) being a statistical technique, its application within ML has become increasingly common. Frequently, papers are reporting the performance of LR under the umbrella of ML algorithms due to them achieving the same objectives of predicting binary outcomes using input features. Additionally, although LR encompasses only a linear combination of risk factors and is unable to model complex relationships, it is insightful to see if simpler models that are a step above clinical risk scores yield the same or similar predictiveness to ML, removing the need for overly complex frameworks and increasing interpretability.

### Risk of Bias and Reporting Quality

The Transparent Reporting of a Multivariable Prediction Model for Individual Prognosis or Diagnosis framework^11^ and the Prediction Model Risk of Bias Assessment Tool^12^ were chosen to assess reporting quality and risk of bias of the included studies. The Transparent Reporting of a Multivariable Prediction Model for Individual Prognosis or Diagnosis framework encompasses a 22‐item checklist to evaluate whether a study is robust and has met standard reporting guidelines including confirmations on the sources of data, interpretation of results, and limitations of the study. Prediction Model Risk of Bias Assessment Tool contains 20 questions categorized into 4 domains: participants, predictors, outcome, and analysis to identify shortcomings and design flaws of a study. Both frameworks were slightly modified to be applicable for ML reporting, as the completion of Transparent Reporting of a Multivariable Prediction Model for Individual Prognosis or Diagnosis‐Artificial Intelligence (AI) and Prediction Model Risk of Bias Assessment Tool‐AI is still pending.^13^ The specific changes are detailed in Tables S2–S5.

## Results

### Search Results

**Figure 1:**
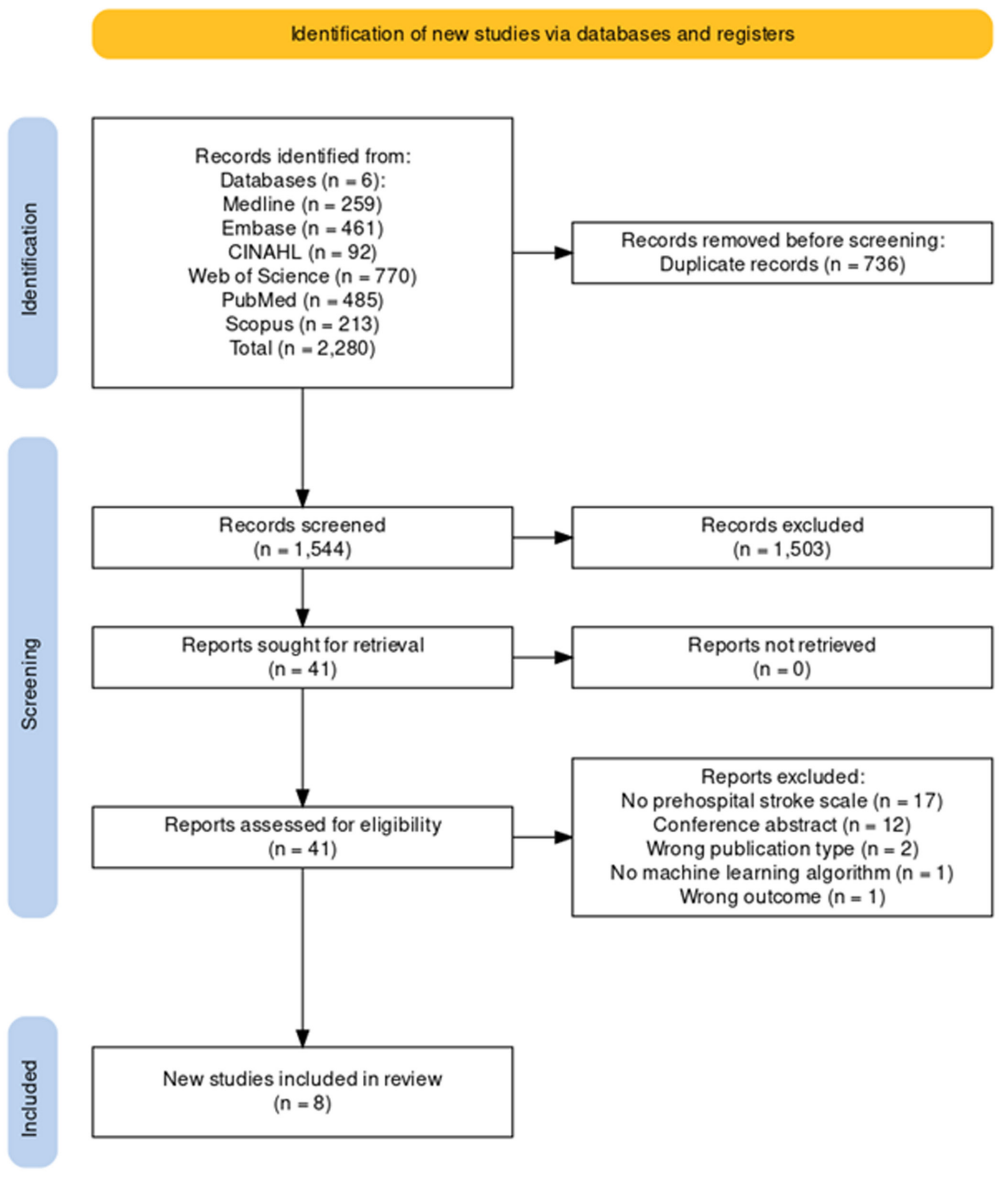
Preferred Reporting Items for Systematic Reviews and Meta‐Analyses 2020 flow diagram displaying selection criteria of the included studies.

The searches returned 2280 articles and 736 duplicates were removed. After screening the title and abstract of the remaining 1544, a total of 41 full texts were retrieved for screening. Eight studies met the inclusion criteria and were included (Figure 1).^14^, ^15^, ^16^, ^17^, ^18^, ^19^, ^20^, ^21^

### Study Characteristics

The characteristics of the 8 included studies are shown in Table 1. These studies included a total of 32 prehospital stroke scales and 21 ML models. Among them, 9 prehospital stroke scales^15^, ^16^, ^18^, ^19^, ^20^, ^21^ and 9 ML models^14^, ^15^, ^16^, ^17^, ^18^, ^19^, ^20^, ^21^ were eligible for meta‐analysis. Included studies were published from 2018^14^ onwards and conducted globally (United States,^16^, ^19^ Japan,^15^, ^20^ China^14^, ^21^ (2 studies each) and 1 each from Hungary^18^ and Taiwan^17^). There was large variation in the total number of participants, from 268^19^ to 19 580^21^. The mean or median age of included participants ranged from 59 years^16^ to 74 years,^15^ with the proportion of women ranging from 36%^21^ to 49%.^17^ The proportion of LVO event rates ranged from 6%^16^ to 50%.^14^ Data on proportion of LVO per occlusion site (anterior versus posterior stroke) was available for only 1 study.^19^ The definitions of LVO were not consistent across the studies. Overall, the definitions of LVO in these studies are based on the occlusion of specific cerebral arteries detected by various imaging modalities, with slight variations in the inclusion/exclusion criteria and arteries considered for LVO classification [Table 1].

**Table 1:** Basic Characteristics of the Included Studies Comparing Machine Learning Models to Prehospital Stroke Scales

Author (year)	Place of recruitment, periods of recruitment	Age mean (SD) or median [interquartile range] years	Sex (male % vs female %)	Total *N* (training data set/test data set)	Data source	LVO *N* (%)	Outcome
Wang (2022)^21^	China, 2016–2021	Training, 70 [60–79] years, test, 71 [60–79] years	Training: 61.8% vs 38.2%, Test: 64% vs 36%	19 580 (15 365/ 4215)	Hospital	5696 (29%)	Anterior or posterior circulation occlusion
Tarkanyi (2022)^18^	Hungary, November 2017–July 2019	68 (13) years	53.8% vs 46.2%	526 (420 /106)	Emergency department	227 (43.2%)	Anterior or posterior circulation occlusion
Hayashi (2021)^15^	Japan, September 2018–September 2020	Training, 74 [65–82] years, test, 73 [61–81] years	Training: 59.6% vs 40.4% Test: 58.6% vs 41.4%	1446 (1156 / 290)	Prehospital	205 (14%)	LVO, ICH, and SAH
Huo (2021)^16^	United States, 2016–2017	59 (n.a.) years	n.a.	1132 (679/453)	Emergency department	67 (6%)	LVO
Sung (2021)^17^	Taiwan, 2016	65.3 (15.6) years	60% vs 49%	1361	Emergency department	443 (31.8%)	Ischemic stroke or TIA
Thomas (2021)^19^	United States, July 2018–June 2019	68 (15) years	53% vs 47%	268	Hospital	84 (30%)*	Anterior or posterior circulation occlusion
Uchida (2021)^20^	Training: Japan; June 2015–March 2018 Test: April 2019–March 2020	Training, 71 (15.4) years, test, 70 (17.4) years	Training: 53.8% vs 46.2% Test: 54.2% vs 45.8%	6305 (3178 / 3127)	Prehospital	520 (16.5%)	LVO
Chen (2018)^14^	China; 2009–2017	67.5 (12.5) years	61.3% vs 38.7%	600 (540/60)	Hospital	300 (50%)	Anterior or posterior circulation occlusion

CT indicates computed tomography; CTA, computed tomography angiography; ICH, intracerebral hemorrhage; LVO, large vessel occlusion; MRA, magnetic resonance angiography; MRI, magnetic resonance imaging; n.a., not available; SAH, subarachnoid hemorrhage; and TIA, transient ischemic attack.

* Only this study reported the proportion of LVO per occlusion site (anterior vs posterior [80 vs 4]). LVO definitions: Wang^21^ and Tarkanyi^18^: occlusion of specific cerebral arteries intracranial internal carotid artery, M1/M2/M3 segments of the middle cerebral artery, anterior cerebral artery, posterior cerebral artery, basilar artery, and vertebral artery based on CT angiography or time‐of‐flight MRA; Hayashi^15^ and Uchida^20^: occlusion of specific cerebral arteries (internal carotid artery, M1 or M2 segments of the middle cerebral artery, and basilar artery) based on CT, MRI, CTA, and MRA; Chen^14^: occlusion of specific cerebral arteries (intracranial ICA, M1/M2 segments of the middle cerebral artery, and basilar artery); Sung^17^: occlusion of cerebral large vessels detected by CTA or MRA, accompanied by low‐ or high‐density areas on CT or diffusion‐weighted MRI; Huo^16^: ischemic stroke, TIA, ICH, subarachnoid hemorrhage, and cerebral venous thrombosis presented within 10 days of symptom onset to a specialized hospital and received CT/MRI examinations; Thomas^19^: examined by a neurologist, had National Institutes of Health Stroke Scale score, and based on CT brain scan.

### Model Development

Details of the ML algorithms are included in Table 2. The most commonly used were RF (n=6^15^, ^16^, ^17^, ^19^, ^20^, ^21^), XGB (n=5^15^, ^16^, ^19^, ^20^, ^21^), and LR (n=4^15^, ^19^, ^20^, ^21^). Two studies^9^, ^18^ chose multiple imputation by chained equations as the method for handling missing data, 2^15^, ^20^ opted for complete case analysis, and 1 paper^17^ replaced missing values with the mean or mode depending on variable type. For prehospital stroke scales, the most commonly used were Pre‐hospital Acute Stroke Severity (n=5^15^, ^16^, ^19^, ^20^, ^21^), NIHSS (n=4^15^, ^16^, ^18^, ^21^), and the Cincinnati Pre‐hospital Stroke Severity Scale (n=4^16^, ^19^, ^20^, ^21^). All prehospital stroke scales used in the included studies were derived from the NIHSS, with 1 study^20^ incorporating additional medical history.

**Table 2:** Model Development Using Machine Learning Algorithms of the Included Studies

Author (year)	Model	Missing values	Software function and version	Total features No.	No. of important features identified
Wang (2022)^21^	RF, LR, XGB, ANN, KNN, AdaBoost, Light GBM, and GBM	No missing values	Python, scikit‐learn, and SPSS	52	17 (total NIHSS total, gaze deviation, LOC, LOC commands, motor left leg, motor left arm, motor right arm, LOC questions, aphasia, motor right leg, facial palsy, dysarthria, age, systolic blood pressure, diastolic blood pressure, AF, and sensory)
Tarkanyi (2022)^18^	LR, elastic net method, and NN	Multiple imputation	SPSS R	39	9 (language, facial palsy, LOC questions, visual field disturbance, gaze palsy and upper limb weakness, AF, chronic heart failure, and white blood cell count)
Hayashi (2021)^15^	RF, LR, XGB, SVM (radial basic function) and SVM (linear)	Complete case analysis	Python 3.7.6 (Scikit‐learn 0.23.2, XGBoost 1.1.1, Pandas 1.1.5, and NumPy 1.19.2)	30	8 (sudden headache, upper limb paralysis, convulsion, sudden impaired consciousness or headache, SBP, arrhythmia, conjugate deviation, and DBP)
Huo (2021)^16^	RF, XGB, KNN, gradient boosting tree, AdaBoost, SVM, linear regression, stochastic gradient descent classifier, Gaussian Naïve Bayes, NN, decision tree, and Gaussian process	No information	Python 3.7.1, scikit‐learn 0.20.0	13	13 (NIHSS items: LOC, LOC questions, LOC commands, gaze, visual fields, facial palsy, arm weakness, leg weakness, limb ataxia, sensory deficit, language/aphasia, dysarthria, extinction/inattention)
Sung (2021)^17^	RF, LR, KNN, CART, SVM, C4.5	Mode and mean imputation	Weka 3.8.3 (for models), Stata 15.1, and R version 3.6.2	23	6 (older age, extremity weakness/neurological symptoms (facial palsy, arm weakness, speech disturbance), triage level of Taiwan Triage and acuity scale (1 or 2), and higher DBP)
Thomas (2021)^19^	RF, LR, XGB, CART	Multiple imputation	Python, scikit‐learn	35	10 (any gaze, any LOC, age, first SBP, first DBP, any extinction/inattention, any aphasia, last known well, any visual, and any face/arm/leg weakness)
Uchida (2021)^20^	RF, LR, XGB	Complete case analysis	Python 3.8.0, JMP 14.0	19	8 (age, headache, arrhythmia, LOC, dysarthria, facial palsy, upper limb paralysis and lower limb paralysis)
Chen (2018)^14^	ANN	1:1 matched	Python, scikit‐learn	27	27 (age, sex, prior antiplatelet therapy, 15 NIHSS items, smoking, hypertension, diabetes, hyperlipidemia, prior stroke/transient ischemic attack, AF, hyperhomocystinemia, coronary artery disease, and family history of cerebrovascular disease)

AF indicates atrial fibrillation; ANN, artificial neural network; CART, classification and regression tree; DBP, diastolic blood pressure; GBM, gradient boosting machine; KNN, K‐nearest neighbors; LOC, level of consciousness; LR, logistic regression; NIHSS, National Institutes of Health Stroke Scale; NN, neural network; RF, random forest; SBP, systolic blood pressure; SVM, support vector machine; and XGB, extreme gradient boosting.

### Model Performance

**Figure 2:**
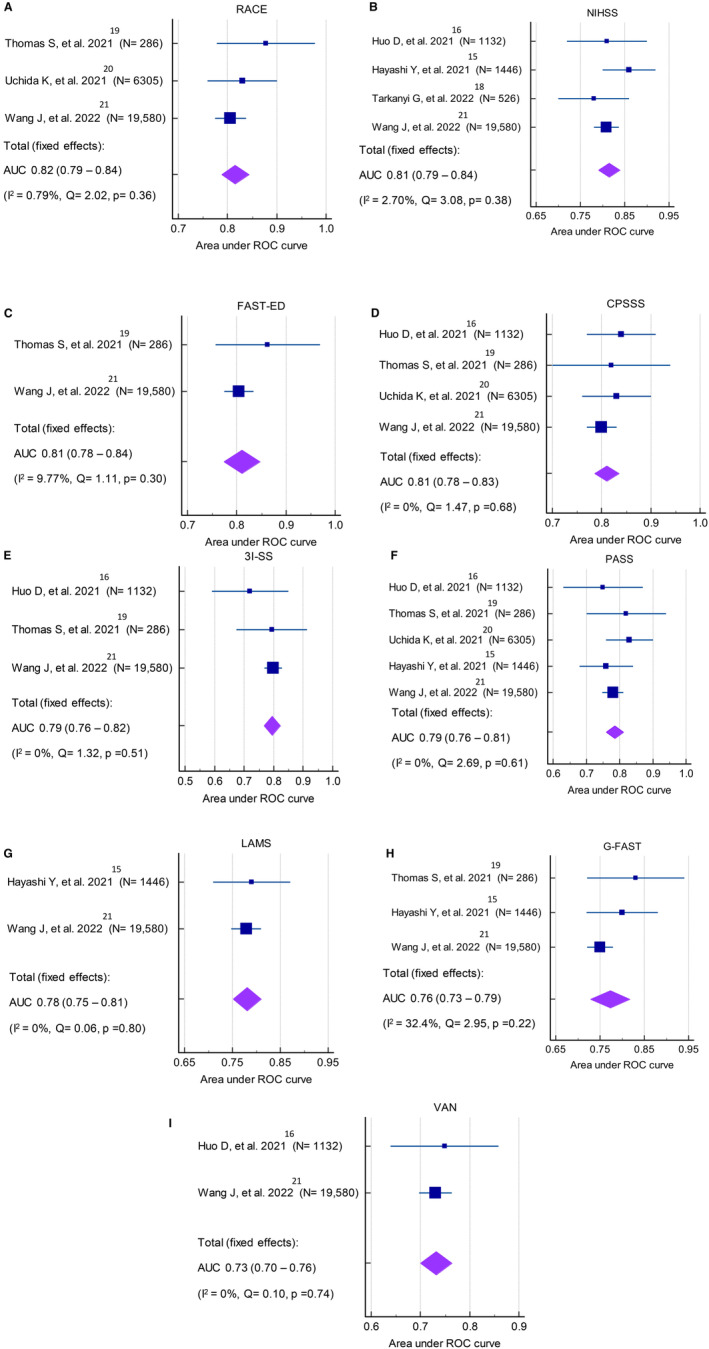
Meta‐analysis of the area under the receiver‐operating curves of prehospital stroke scale predicting large vessel occlusion. **A,** Rapid Arterial Occlusion Evaluation (RACE); **B,** National Institutes of Health Stroke Scale (NIHSS); **C,** Facial palsy, Arm weakness, Speech changes, Time, Eye deviation, Denial/neglect (FAST‐ED); **D,** Cincinnati Pre‐hospital Stroke Severity Scale (CPSSS). **E,** 3‐Item Stroke Scale (3I‐SS); **F,** Pre‐hospital Acute Stroke Severity (PASS); **G,** Los Angeles Motor Scale (LAMS); **H,** Gaze‐face‐arm‐speech‐time (G‐FAST); **I,** Vision, Aphasia, Neglect (VAN). AUC indicates area under the curve; and ROC, receiver operating characteristic.

**Figure 3:**
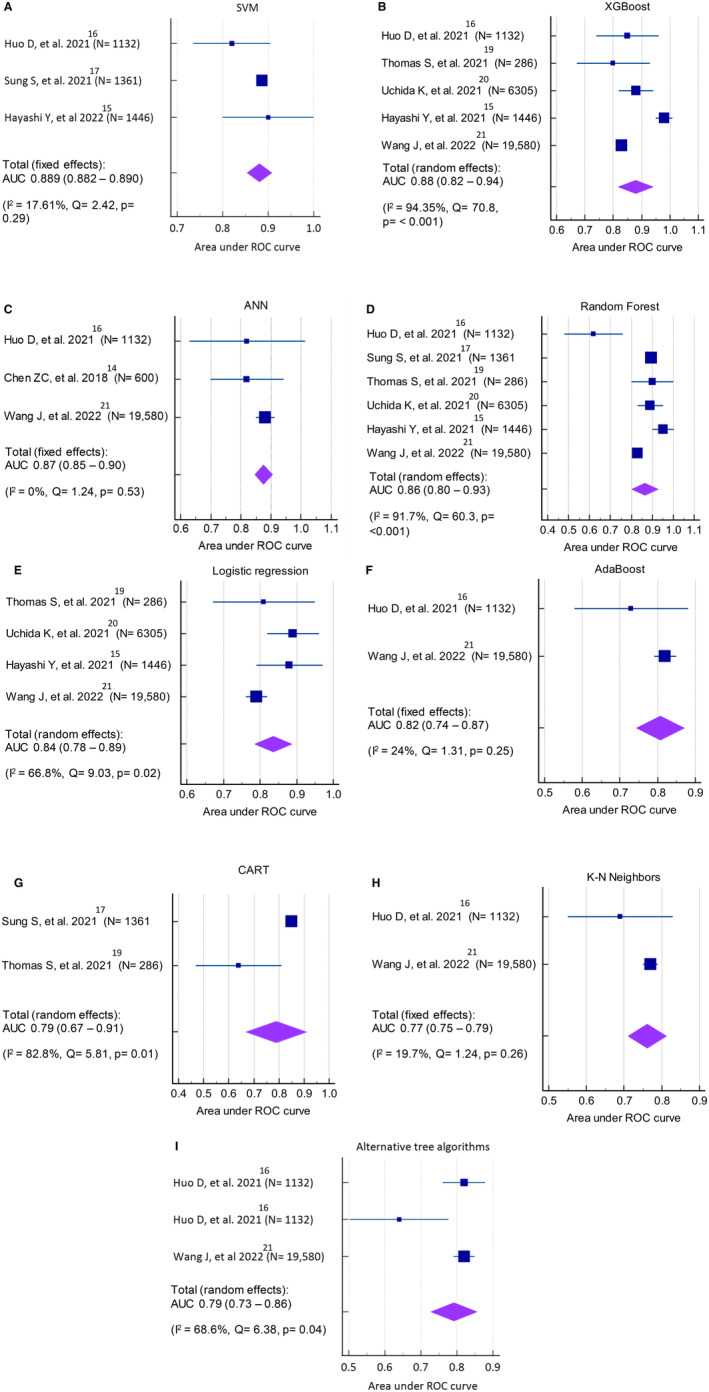
Meta‐analysis of the area under the receiver‐operating curves of machine learning models predicting large vessel occlusion. **A,** Support vector machine (SVM); **B,** Extreme gradient boosting (XGBoost); **C,** Artificial neural network (ANN); **D,** Random forest (RF); **E,** Logistic regression (LR); **F,** AdaBoost; **G,** Classification and regression tree (CART); **H,** K‐nearest neighbors (KNN); **I,** Alternative tree algorithms. AUC indicates area under the curve; and ROC, receiver operating characteristic.

**Figure 4:**
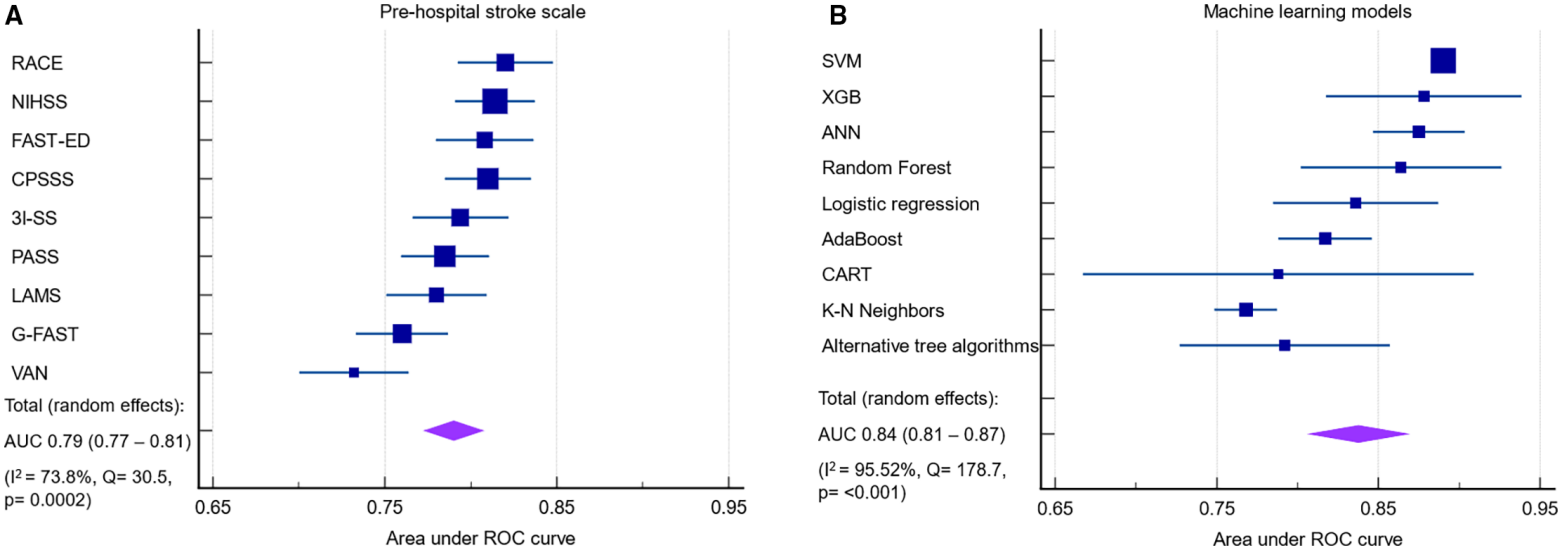
Meta‐analysis of all the area under the receiver‐operating curves of prehospital stroke scale and machine learning models predicting large vessel occlusion. **A,** prehospital stroke scale; **B,** Machine learning models. ANN indicates artificial neural network; AUC, area under the curve; CART, classification and regression tree; CPSSS, Cincinnati Pre‐hospital Stroke Severity Scale; FAST‐ED, Facial palsy, Arm weakness, Speech changes, Time, Eye deviation, Denial/neglect; G‐FAST, gaze‐face‐arm‐speech‐time; KNN, K‐nearest neighbors; LAMS, Los Angeles Motor Scale; NIHSS, National Institutes of Health Stroke Scale; PASS, Pre‐hospital Acute Stroke Severity; RACE, Rapid Arterial Occlusion Evaluation; ROC, receiver operating characteristic; SVM, support vector machine; VAN, Vision, Aphasia, Neglect; XGB, extreme gradient boosting; and 3I‐SS, 3‐Item Stroke Scale.

The pooled AUC performance of the stroke scales and ML models for LVO used by the included studies are shown in Figures 2A, 2B, 3A, 3B and 4. Both Rapid Arterial Occlusion Evaluation and NIHSS had the highest pooled AUC of any prehospital stroke scale (Rapid Arterial Occlusion Evaluation, 0.82 [95% CI, 0.79–0.84]; *I* ^2^: 0.79%) and NIHSS, 0.81 [95% CI, 0.79–0.84]; *I* ^2^: 2.70%)]. The AUC range for prehospital stroke scales was from 0.73 to 0.82. Of the ML models, both Support Vector Machine and XGB had the highest pooled AUC of any ML models (Support Vector Machine, 0.89 [95% CI, 0.88–0.89]; *I* ^2^: 17.61%) and XGB, 0.88 [95% CI, 0.82–0.94]; *I* ^2^: 94.35%)]. The AUC for LVO ranges across all ML models was 0.77 to 0.89. Overall, the pooled AUC of all prehospital stroke scales was (0.79 [95% CI, 0.77–0.81]; *I* ^2^: 73.8%) whereas the pooled AUC of all ML models was (0.84 [95% CI, 0.81–0.87]; *I* ^2^: 95.52%) (Figure 4). Moreover, 2 studies tested their ML models on intracerebral hemorrhage and subarachnoid hemorrhage,^15^, ^20^ but only 1^15^ reported the 95% CI of the AUC. Therefore, meta‐analyses were not performed. The AUC of intracerebral hemorrhage included 3 ML models: XGB: 0.86 (95% CI, 0.82–0.91), RF: 0.82 and LR: 0.82, whereas the AUC of subarachnoid hemorrhage had 3 ML models: XGB: 0.92 (95% CI, 0.87–0.97), RF: 0.85 and LR: 0.87.

**Figure 5:**
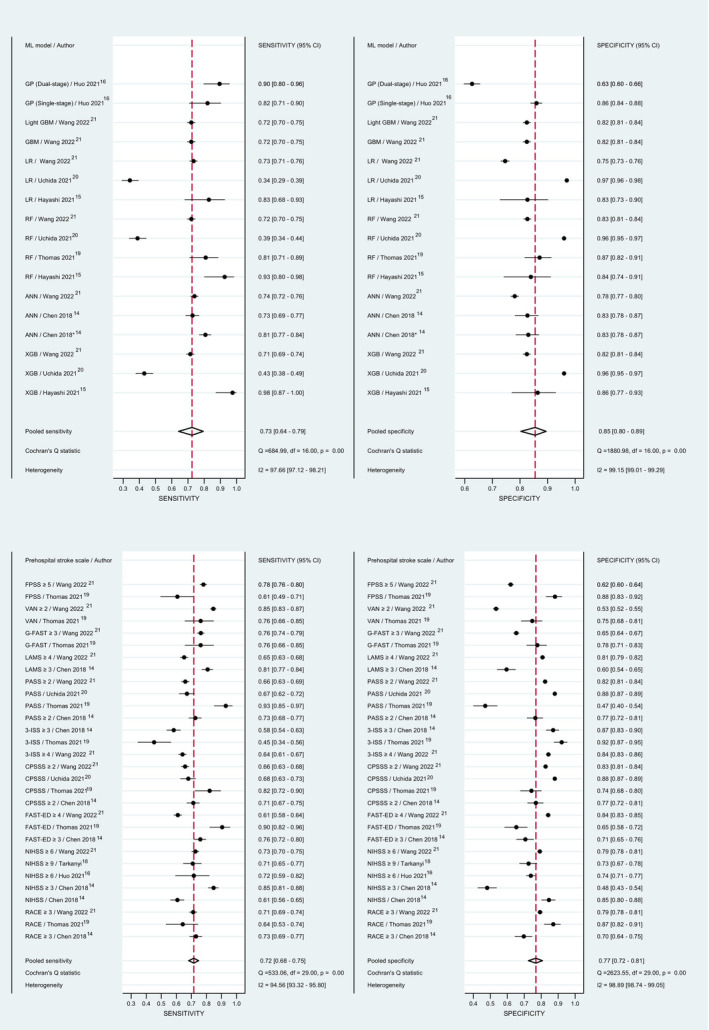
A forest plot demonstrating the individual and pooled sensitivity and specificity of the included machine learning studies (A) and a forest plot demonstrating the individual and pooled sensitivity and specificity of the included prehospital stroke scales studies (B). ANN indicates artificial neural network; CPSSS, Cincinnati Pre‐hospital Stroke Severity Scale; FAST‐ED, Facial palsy, Arm weakness, Speech changes, Time, Eye deviation, Denial/neglect; FPSS, finnish prehospital stroke scale; GBM, gradient boosting machine; G‐FAST, gaze‐face‐arm‐speech‐time; GP, Gaussian process; LAMS, Los Angeles Motor Scale; LR, logistic regression; NIHSS, National Institutes of Health Stroke Scale; PASS, Pre‐hospital Acute Stroke Severity; RACE, Rapid Arterial Occlusion Evaluation; RF, random forest; VAN, Vision, Aphasia, Neglect; XGB, extreme gradient boosting; and 3I‐SS, 3‐Item Stroke Scale.

**Figure 6:**
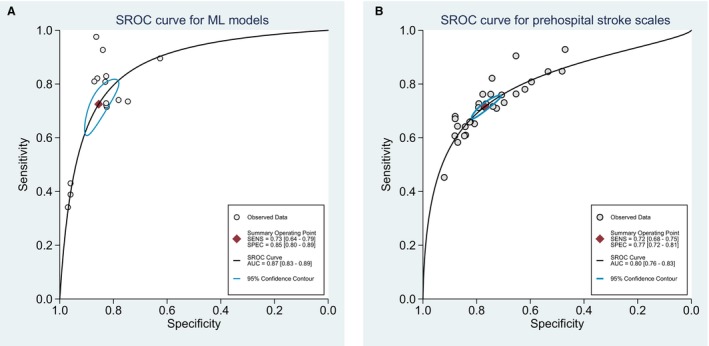
Summary ROC curve of machine learning models (A) and summary ROC curve of prehospital stroke scales (B). AUC indicates area under the curve; ML, machine learning; SENS, sensitivity; SPEC, specificity; and SROC, summary receiver operating characteristic.

In addition, considering the challenges associated with comparing AUCs across studies, which involve variations in sample populations, measurement accuracy, covariate selection, pooled sensitivity, specificity, and SROC curve for all available prehospital stroke scales and ML models were performed and are shown in Figures 5A, 5B, and 6A, 6B.

For all prehospital stroke scales, the sensitivity ranged from 0.45 to 0.93, and specificity ranged from 0.47 to 0.92 in 7 of the included studies (Figure 5B).^14^, ^15^, ^16^, ^18^, ^19^, ^20^, ^21^ The pooled sensitivity of the included studies (*n*=7) was 0.72 (95% CI, 0.68–0.75, *I* ^2^=94.56%) and specificity was 0.77 (95% CI, 0.72–0.81, *I* ^2^=98.74%). The summary ROC curve for prehospital stroke scales yielded a high AUC value (0.80 [95% CI, 0.76–0.83]) (Figure 6B). When stratified by the type of scale, only NIHSS, Cincinnati Pre‐hospital Stroke Severity Scale, and Pre‐hospital Acute Stroke Severity were eligible for pooled sensitivity and specificity and SROC curve due to the low number of studies using other scales (Figures S1, S2A,B and S3A,B).

For all ML, the sensitivity ranged from 0.34 to 0.98, and specificity ranged from 0.63 to 0.97 in 6 of the included studies (Figure 5A).^14^, ^15^, ^16^, ^19^, ^20^, ^21^ The pooled sensitivity of the included studies (*n*=6) was 0.73 (95% CI, 0.64–0.79, *I* ^2^=97.66%) and specificity was 0.85 (95% CI, 0.63–0.97, *I* ^2^=99.15%). The summary ROC curve for ML yielded a high AUC value (0.87, 95% CI, 0.83–0.89; Figure 6A). When stratified by the ML model, only those using RF were eligible for pooled sensitivity and specificity and SROC curve due to the low number of studies using other models (Figure S4A,B).

### Variable Importance

**Figure 7:**
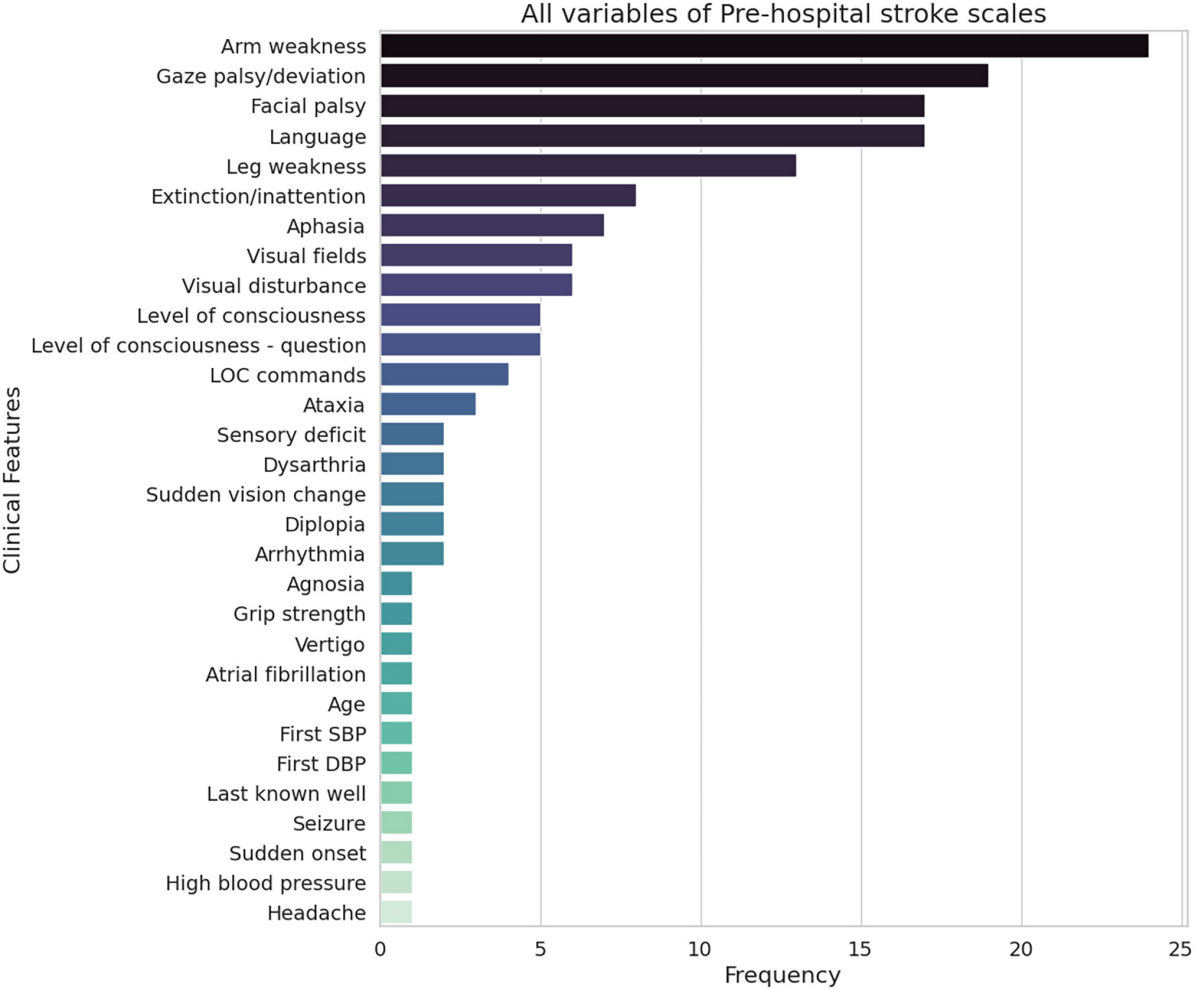
All variables included in prehospital stroke scales of the included studies along with the count of the number of stroke scales that used the variable. DBP indicates diastolic blood pressure; LOC, level of consciousness; and SBP, systolic blood pressure.

Thirty‐two prehospital stroke scales were identified in the included studies, with many different variables (mainly clinical neurological symptoms) incorporated into the scales. Arm weakness (n=24/32), horizontal gaze deviation (n=19/32), facial palsy, and language (both n=17/32) were the most commonly selected variables in the 32 prehospital stroke scales (Figure 7).

**Figure 8:**
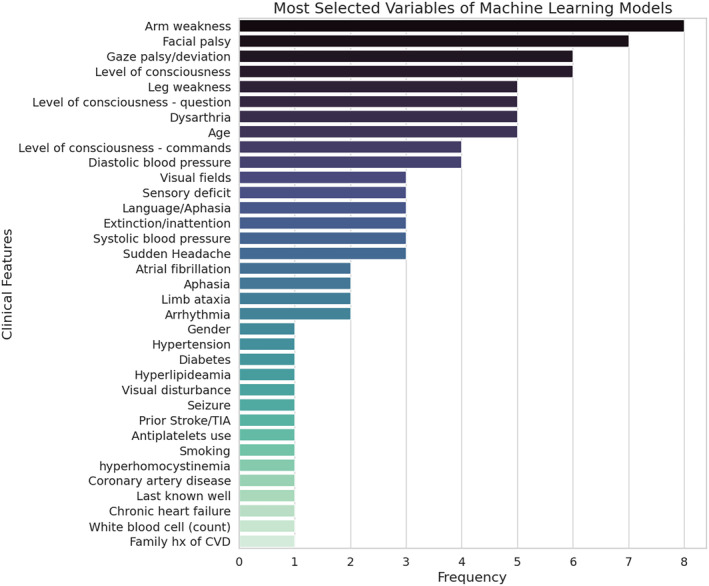
Selected variables in machine learning models of included studies (most important variables reported in the included studies). CVD indicates cerebrovascular disease; and TIA, transient ischemic attack.

Twenty‐one ML models were identified in the included studies. The most commonly selected variables were arm weakness (n=8/21), facial palsy (n=7/21), horizontal gaze deviation, and level of consciousness (both n=6/21) (Figure 8).

### Risk of Bias

Of the 8 included studies, 2 were categorized as low risk of bias,^15^, ^18^ 4 as high risk,^16^, ^17^, ^19^, ^20^ and 2 as unclear risk of bias^14^, ^21^ (Figure S5). All studies had low risk of bias for the participants, predictors, and outcome sectors; potential bias arose in relation to the analysis sector. A breakdown of the assessment of each study compared with the specific framework criteria is presented in Tables S2 and S3. The most common cause for high risk of bias was related to the number of participants with the outcome. Other factors included handling of continuous and categorical variables, missing data methods, and the measurement of model or scale performance.

### Reporting Quality

The proportion of adherence to the Transparent Reporting of a Multivariable Prediction Model for Individual Prognosis or Diagnosis checklist is displayed in Figure S6. Only 1 study^14^ did not meet the criterion of “good” adherence (≥70%). All items of the checklist that were not met mainly occurred in the “results” section (Tables S4 and S5). The least adhered to element of the checklist was reporting performance measures with lack of appropriate detail reported, including CIs, *P* values, and ranges. Other elements identified included the lack of specifying the number of participants and outcomes, as well as a clear description of the characteristics of participants.

## Discussion

This systematic review and meta‐analysis compared the performance of prehospital stroke scales and ML models for detecting LVO. The findings demonstrated that Rapid Arterial Occlusion Evaluation and NIHSS had the highest pooled AUC of any prehospital stroke scales, and XGB and Support Vector Machine had the highest pooled AUC of any ML models. Both prehospital stroke scales and ML models had a variable accuracy in predicting LVO. The pooled sensitivity, specificity, and SROC curve of the included studies were slightly higher in ML models than prehospital stroke scale. There were higher heterogeneity of pooled AUC, sensitivity, and specificity in the ML models compared with prehospital stroke scales, mainly due to methodological differences, small sample size, lower outcome rates, and high risk of bias. However, despite the high discrimination or performance of these ML or prehospital stroke scales, this could raise a question about their validity and generalizability. In addition, the sensitivity‐specificity tradeoff in pooled sensitivity, specificity, and SROC curves poses a common challenge in diagnostic testing. Increasing sensitivity may lead to a decrease in specificity and vice versa. This tradeoff often results in substantial heterogeneity due to variations in tradeoff preferences across studies. Some scales and ML models might prioritize sensitivity over specificity, whereas others may emphasize specificity at the expense of sensitivity. Therefore, it is crucial to carefully consider the clinical context and the goals of the diagnostic test, ensuring alignment with specific clinical requirements and objectives.

Most of the included studies were single‐center retrospective data sets. Although training and internal validation processes were often used, there were few data collected prospectively and there is no direct trial evidence. Consequently, although performance of the standard and ML models was high (AUC>0.8), it is important to note that this may not be what is achieved in a real world prehospital setting.

The RACECAT (Direct Transfer to an Endovascular Center Compared to Transfer to the Closest Stroke Center in Acute Stroke Patients With Suspected Large Vessel Occlusion) trial^22^ and Stockholm Stroke Triage Study,^23^ both large prehospital studies of LVO, showed lower prehospital positive predictive values for LVO (50% and 41%, respectively) than the included studies. Thus, the results of the included studies should be interpreted with caution when using predictive scores to triage prehospital stroke patients, as the performance and implementation of these scores in real‐world settings may be lower than reported in this review.

However, there are a number of possible explanations for this difference. The RACECAT trial and Stockholm Stroke Triage Study were conducted in prehospital settings, whereas the included studies were mainly conducted in hospital settings and included clinicians with experience in NIHSS assessment and with availability of medical records. These differences may explain why the performance of predictive scores in the RACECAT trial and Stockholm Stroke Triage Study were lower than the performance of predictive scores in the included studies.

Potential justification for enhanced performance rests on ML being able to incorporate more complex relationships between variables that moves beyond the traditional linear combination. Through improving prediction tools and obtaining more influential risk factors, a number of benefits are possible including increasing treatment accessibility, minimizing brain damage, and achieving better clinical outcomes. The analysis domain was identified as the most common source of bias, which can be attributed to a small number of participants with the desired outcome, challenges in handling both continuous and categorical variables during analysis, approaches used for addressing missing data, and the measurement of model or scale performance. These factors can potentially introduce bias and impact the validity and generalizability of the study findings. Similarly, the majority of the included studies demonstrated borderline “good” (≥70%) quality reporting, with 1 study showing poor adherence to reporting standards. Several elements were identified as suboptimal, including specifying the number of participants and outcomes, as well as a clear description of the characteristics of the study participants. Additionally, the reporting of performance measures lacked appropriate detail, such as the inclusion of CIs, *P* values, and ranges. These shortcomings in reporting standards limit the transparency and reproducibility of the study findings.

Although all of the included studies predict LVO, there is insufficient detail on whether the data used encompass anterior and posterior vessel occlusions. This is important as any model developed based on only one, anterior or posterior, may not be applicable to cases of the other. This review identified that only 1 study^19^ reported the proportion of anterior and posterior circulation occlusion.

Given reports surrounding the importance of appropriate missing data handling^24^ and given that it was one of the factors of induced potential bias, reliance on applying robust and suitable methods is paramount in data preprocessing. By using incorrect methods, a range of problems can occur including invalid inferences and particularly in ML, the creation of distorted relationships between variables. In many cases, the general recommendation is multiple imputation,^25^ but is data dependent.

As ML applications have begun achieving promising results, barriers to implementation in health care settings are frequently discussed.^26^ In this review, no study externally validated their model. Bias detection, generalization, and reliability are a number of critical reasons to validate a ML model on unseen, ideally diverse data. Another barrier stemming from the models in this review is the lack of code provided in publications to replicate the included studies. Of the 8 included studies, only 1^17^ provided source code information on accessing the data; however, 2^20^, ^21^ stated that both could be requested. Without specific and detailed algorithm development information, the model is unlikely to be reliable and valid for implementation. This in turn contributes a substantial barrier to real‐world implementation, which draws skepticism from health care professionals. Without major understanding of the explainability and interpretability of ML models, health care professionals are often dubious regardless of a model's notable performance. Transparency is a focus of many current ML applications to gain trust by the user and provider.^27^

A final barrier facing clinical implementation of ML surrounds whether the models are feasible in the tested setting. Prehospital settings are often fast paced and resource limited, which justifies why many of the current prehospital stroke scales comprise a small number of variables. Although ML has the potential to embed complex interactions between variables, having a tool that requires many collected features may limit their flexibility in a prehospital setting. Integration of ML models for LVO detection in prehospital care systems or mobile applications is possible, but nonetheless requires extensive validation and testing to ensure their reliability and safety.

### Strengths and Limitations

This review followed rigorous methodology ensuring that all relevant studies were identified and included. Two independent reviewer authors systematically completed abstract screening, full‐text screening, data extraction, and risk of bias assessment, enhancing the credibility and reliability of our findings. In addition, to ensure comprehensive coverage of all relevant studies and to avoid the risk of publication bias, we conducted searches in multiple databases, thereby implementing a wide‐ranging and inclusive search strategy. Moreover, the meta‐analysis provided a more quantitative summary of the overall discrimination metrics of the ML models and prehospital stroke scales, further enhancing the robustness of our conclusions.

Although this is the first comprehensive systematic review comparing ML methods to current stroke scales in a prehospital setting for detection of LVO, there remain a number of limitations within this work. Within this review, LVO was the only outcome explored as ischemic strokes are more common and, hence affect a greater number of people.

Another limitation within this paper surrounds the difficulty in drawing concrete conclusions on predictive variables learned from the ML approaches. Not every study included the same variables, and therefore commenting on which variables were the most predictive of LVO is inappropriate. A single study may have used a variable that is highly predictive; however, multiple studies may have all found a consistent predictive variable with lower influence, hence commenting on the influence of variables from ML models should be noted with caution.

The studies included in this review did not perform external validation on their ML models. Therefore, although in the data sets tested they outperform clinical risk scores, this could be attributed to the models being developed on the same data. Without external validation in an unseen cohort, distinction as to whether the ML models are overfitting to the training data is unclear. Finally, the heterogeneity and variation of the clinical populations and settings of the included studies are a limitation of this review. It is therefore important to interpret the results with caution, as the results of the review may not be generalizable to all prehospital stroke patients.

### Implication for Future Research

Future studies should aim to include more homogeneous populations and prehospital settings and could focus on improving prehospital ML models for LVO detection by refining and optimizing algorithms, incorporating additional relevant features, and exploring ensemble methods to enhance accuracy and robustness. Validation studies should be conducted on larger and more diverse populations of patients with suspected stroke, including external validation in different data sets and geographic regions to assess performance in diverse stroke populations. Additionally, research should concentrate on methods to enhance interpretability and explainability of ML models used in prehospital settings, increasing trust and acceptance among prehospital providers. Feasibility studies investigating the impact of implementing ML models scores in prehospital practice and integrating ML models into emergency medical services workflows could help assess their effect on patient outcomes and resource use. However, implementing ML models for prehospital stroke triage in real‐world settings may be challenging due to their complexity, cost, and training requirements for prehospital providers. Also, despite some of the ML models and scales not being widely used in prehospital settings, the findings of this review can still improve prehospital stroke triage. The NIHSS and RACE scores may be particularly useful for identifying patients with LVO in hospital settings, but further research is needed to develop and evaluate predictive scores specifically designed for prehospital use. Lastly, international collaborations and data sharing among research institutions and health care providers could help build larger and more diverse data sets for training and validating machine learning models. Therefore, by addressing these implications in future research, the prehospital field can advance understanding of the role of prehospital ML models in improving early detection and management of LVO in patients with suspected stroke. Facial recognition AI technology has the potential to revolutionize stroke detection. Two previous studies have shown that facial recognition can detect stroke with an accuracy of over 95%.^28^, ^29^ One study developed a ML model (Support Vector Machine, RF, and Bayes) that was able to detect facial stroke recognition with an accuracy ranging from 95.5% to 100%, whether used by patients or health care providers.^28^, ^29^ The model was able to identify the subtle changes in facial features associated with stroke, such as the asymmetry index, which was calculated using slope, area ratio, and distance ratio between the left and right eye and mouth reflecting facial palsy. However, the model was trained on a small data set of 69 images of faces with and without stroke, with no information on the type of stroke or LVO. Another study developed an Android mobile app that used AI to detect facial palsy, arm strength, and speech abnormalities, with high accuracy in detecting stroke (AUC = 0.95).^27^ These studies demonstrate that AI has the potential to be a valuable tool for stroke detection. The development of AI‐powered stroke detection tools is still in its early stages, but the results of these studies appear promising.

## Conclusions

The systematic review and meta‐analysis suggest that ML methods may have the potential to improve the detection of LVO in the prehospital setting, compared with prehospital stroke scales. Despite the slightly higher discrimination in ML models compared with prehospital stroke scales, the potential benefits of this improved discrimination need to be carefully weighed against the additional resources required for implementing these models in clinical practice. However, the studies included in this review explored populations with limited diversity regarding the proportion of anterior or posterior circulation occlusions, which limits the generalizability of the findings to other populations and applicability. Despite these limitations, the findings of this review are encouraging and suggest that ML may be a valuable tool for improving prehospital stroke triage. Future research should focus on developing and validating ML models that are specifically designed for use in the prehospital setting and that are generalizable to diverse populations. Additionally, feasibility studies and real‐world application studies are needed to assess the feasibility and impact of implementing ML models for LVO detection in prehospital settings. By addressing these areas, we can advance the integration of ML into prehospital stroke care and potentially improve detection and outcomes for patients with suspected stroke.

## Sources of Funding

Muath Alobaida is funded by a PhD studentship from King Saud University.

## Disclosures

Yalin Zheng reports consultancy work for F. Hoffmann‐La Roche AG and AIMs, which are outside the scope of the current study. Gregory Y. H. Lip is a consultant and speaker for BMS/Pfizer, Boehringer Ingelheim, Anthos, and Daiichi‐Sankyo; no fees are received personally. Deirdre A. Lane received investigator‐initiated educational grants from Bristol‐Myers Squibb and Pfizer, has been a speaker for Bayer, Boehringer Ingelheim, and Bristol‐Myers Squibb/Pfizer and has consulted for Bristol‐Myers Squibb and Boehringer Ingelheim; all outside the submitted work. SLH has received grant funding from Bristol‐Myers Squibb outside of the submitted work. The remaining authors have no disclosures to report.

## Supporting information

Data S1
Tables S1–S4
Figures S1–S6
Reference 30
