# Supplementary material for: Systematic Review and Meta‐Analysis of Prehospital Machine Learning Scores as Screening Tools for Early Detection of Large Vessel Occlusion in Patients With Suspected Stroke
Source: J Am Heart Assoc. 2024 Jun 11;13(12):e033298. doi: 10.1161/JAHA.123.033298 (PMC11255760; doi:10.1161/JAHA.123.033298)
Supplement: Supplementary file 1 — Data S1 Tables S1–S4 Figures S1–S6 Reference 30 [file JAH3-13-e033298-s001.docx]

Supplemental Material

**Table S1**. Search strategy for all databases from inception to 10^th^ October 2023.

| No. | Database | Results |
| --- | --- | --- |
| 1 | Ovid MEDLINE(R) ALL | 259 |
| 2 | PubMed | 485 |
| 3 | The Cumulative Index to Nursing and Allied Health Literature (CINAHL) | 92 |
| 4 | Web of Science | 770 |
| 5 | Scopus | 213 |
| 6 | Ovid Embase Classic+Embase | 461 |
| Total | Ovid MEDLINE, PubMed, CINAHL, WOS, Scopus and Embase | 2280 |
| Duplicates | Ovid MEDLINE, PubMed, CINAHL, WOS, Scopus and Embase | 736 |
| Total | Ovid MEDLINE, PubMed, CINAHL, WOS, Scopus and Embase | 1544 |

| **Ovid MEDLINE(R) ALL <1946 to October, 2023>** | | |
| --- | --- | --- |
| # | Query | Results |
| 1 | artificial intelligence.mp. or Artificial Intelligence/ | 47,682 |
| 2 | Machine learning.mp. or Artificial Intelligence/ or Machine Learning/ or Algorithms/ | 367,420 |
| 3 | (AI or ML).mp. | 886,423 |
| 4 | isch?emic stroke.mp. or Stroke/ or Ischemic Stroke/ or stroke.mp. | 154,729 |
| 5 | (thrombectomy or Endovascular thrombectomy or mechanical thrombectomy or large vessel occlusion*).mp. | 18151 |
| 6 | (prehospital or pre-hospital or pre hospital or out-of-hospital or out of hospital or paramedic or emergency medical service* or emergency medical technician or pre-hospital care or pre hospital care or prehospital care or allied health care or ambulance or ambulance staff or helicopter or triage).mp. | 104,211 |
| 7 | 1 or 2 or 3 | 1,242,764 |
| 8 | 4 or 5 | 358,271 |
| 9 | 6 and 7 and 8 | 259 |

| **PubMed: National Library of Medicine** | | |
| --- | --- | --- |
| # | Query | Results |
| 1 | ((algorithms[MeSH Terms]) OR (ai artificial intelligence[MeSH Terms])) OR (machine learning[MeSH Terms]) | 404,764 |
| 2 | ((((((brain ischemia[MeSH Terms]) OR (stroke[MeSH Terms])) OR (thrombectomy[MeSH Terms])) OR (large artery occlusion)) OR (large vessel occlusion))) OR (brain ischaemia[Mesh Terms]) | 229,010 |
| 3 | (((((prehospital) OR (pre-hospital)) OR (emergency)) OR (paramedic)) OR (triage)) OR (allied health personnel*) | 1,295,765 |
| 4 | out-of-hospital OR out hospital OR pre hospital OR ambulance OR EMS OR emergency medical service* OR pre-hospital OR prehospital OR pre hospital care OR helicopter OR air ambulance | 725,323 |
| 5 | #3 OR #4 | 1,826,224 |
| 6 | #1 AND #2 AND #5 | 485 |

| **Database: The Cumulative Index to Nursing and Allied Health Literature (CINAHL) Friday, 10^th^ October 2023** | | |
| --- | --- | --- |
| # | Query | Results |
| 1 | (((MH "Algorithms") OR (MH "Artificial Intelligence+/MT/CL")) OR ((machine AND learning) OR (artificial AND intelligence)) OR ((MH "Artificial Intelligence+") OR (MH "Machine Learning+"))) | 73,429 |
| 2 | (((MH "Cerebral Ischemia+") OR "brain ischemia") OR ((MH "Stroke") OR (MH "Cerebral Ischemia, Transient") OR (MH "Ischemic Stroke") OR (MH "Cerebral Ischemia") OR (MH "Stroke Patients") OR (MH "Cerebrovascular Circulation") OR "stroke or cerebrovascular accident or cva or cerebral vascular event or cve or transient ischaemic attack or tia") OR thrombectomy OR ("large artery occlusion") OR ("large vessel occlusion")) | 100,716 |
| 3 | ((prehospital OR (pre AND hospital) OR pre-hospital OR (out AND of AND hospital) OR (non AND hospital AND setting) OR (prehospital AND care) OR (emergency AND medical AND services) OR (prehospital AND service)) OR (paramedic OR ems OR (emergency AND medical AND service) OR prehospital OR pre-hospital OR ambulance OR (emergency AND medical AND technician) OR emt) OR triage OR ((MH "Allied Health Personnel") OR "allied health personnel")) | 161,093 |
| 4 | S1 AND S2 AND S3 | 92 |

| **Web of Science Core Collection** | | |
| --- | --- | --- |
| # | Query | Results |
| 1 | ((ALL=(algorithms )) OR ALL=(artificial intelligence)) OR ALL=(machine learning) | 2,909,153 |
| 2 | (((((((ALL=(brain ischemia)) OR ALL=(brain ischaemia)) OR ALL=(stroke)) OR ALL=(thrombectomy)) OR ALL=(large artery occlusion)) OR ALL=(large vessel occlusion)) OR ALL=(Ischaemic stroke )) OR ALL=(Ischemic stroke) | 762,103 |
| 3 | (((((ALL=(prehospital)) OR ALL=(pre-hospital)) OR ALL=(emergency)) OR ALL=(paramedic)) OR ALL=(triage)) OR ALL=(allied health*) | 669,059 |
| 4 | #1 AND #2 AND #3 | 770 |

| Database: Scopus ( 10^th^ October 2023) | | |
| --- | --- | --- |
| # | Query | Results |
| 1 | TITLE-ABS-KEY(((ai OR ml) .mp.)) OR (TITLE-ABS-KEY (artificial AND intelligence.mp. OR artificial AND intelligence/)) OR (TITLE-ABS-KEY (machine AND learning.mp. OR machine AND learning/ OR algorithms)) | 969,254 |
| 2 | ( ( TITLE-ABS-KEY( thrombectomy ) ) OR ( TITLE-ABS-KEY( endovascular AND thrombectomy ) ) OR ( TITLE-ABS-KEY( mechanical AND thrombectomy ) ) ) OR ( TITLE- ABS-KEY( stroke/ OR isch?mic AND stroke/ ) ) OR ( TITLE-ABS-KEY(large AND vessel AND occlusion )) | 514,162 |
| 3 | ( TITLE-ABS-KEY ( ambulance AND staff ) ) OR ( TITLE-ABS-KEY ( allied AND health AND care ) ) OR ( TITLE-ABS-KEY ( prehospital AND care ) ) OR ( TITLE-ABS-KEY( pre AND hospital AND care ) ) OR ( TITLE-ABS-KEY( pre-hospital AND care ) ) OR ( TITLE-ABS-KEY( emergency AND medical AND technician ) ) OR ( TITLE-ABS-KEY( emergency AND medical AND service* ) ) OR ( TITLE-ABS-KEY( out AND of AND hospital ) ) OR ( TITLE-ABS-KEY( pre AND hospital ) ) OR ( TITLE-ABS-KEY( prehospital OR pre-hospital OR out-of-hospital OR paramedic OR ambulance OR helicopter OR triage OR helicopter OR ems ) ) | 733,526 |
| 4 | #1 AND #2 AND #3 | 213 |

| Ovid Embase Classic+Embase 1947 to 2023 October 10^th^ | | |
| --- | --- | --- |
| # | Query | Results |
| 1 | artificial intelligence.mp. or Artificial Intelligence/ | 51,967 |
| 2 | Machine learning.mp. or Artificial Intelligence/ or Machine Learning/ or Algorithms/ | 360,328 |
| 3 | (AI or ML).mp. | 1,393,473 |
| 4 | isch?emic stroke.mp. or Stroke/ or Ischemic Stroke/ or stroke.mp. | 544,685 |
| 5 | (thrombectomy or Endovascular thrombectomy or mechanical thrombectomy or large vessel occlusion*).mp. | 38,645 |
| 6 | (prehospital or pre-hospital or pre hospital or out-of-hospital or out of hospital or paramedic or emergency medical service* or emergency medical technician or pre- hospital care or pre hospital care or prehospital care or allied health care or ambulance or ambulance staff or helicopter or triage).mp. | 112,270 |
| 7 | 1 or 2 or 3 | 1,733,788 |
| 8 | 4 or 5 | 564,423 |
| 9 | 6 and 7 and 8 | 461 |

| **Table S2. PROBAST questions for risk-of-bias assessment** | |
| --- | --- |
| **Participants** | 1.1 Were appropriate data sources used, e.g., cohort, RCT, or nested case-control study data? |
|  | 1.2. Were all inclusions and exclusions of participants appropriate? |
| **Predictors** | 2.1. Were predictors defined and assessed in a similar way for all participants? |
|  | 2.2. Were predictor assessments made without knowledge of outcome data? |
|  | 2.3. Are all predictors available at the time the model is intended to be used? |
| **Outcome** | 3.1. Was the outcome determined appropriately? |
|  | 3.2. Was a prespecified or standard outcome definition used? |
|  | 3.3. Were predictors excluded from the outcome definition? |
|  | 3.4. Was the outcome defined and determined in a similar way for all participants? |
|  | 3.5. Was the outcome determined without knowledge of predictor information? |
|  | 3.6. Was the time interval between predictor assessment and outcome determination appropriate? |
| **Analysis** | 4.1 Were there a reasonable number of participants with the outcome? |
|  | 4.2. Were continuous and categorical predictors handled appropriately? |
|  | 4.3 Were all enrolled participants included in the analysis? |
|  | 4.4. Were participants with missing data handled appropriately? |
|  | 4.5. Was selection of predictors/ hyperparameter based on univariable analysis avoided? |
|  | 4.6. Were complexities in the data (e.g., censoring, competing risks, sampling of control participants) accounted for appropriately? |
|  | 4.7. Were relevant ML model performance measures evaluated appropriately? |
|  | 4.8. Were model overfitting, underfitting, and optimism in model performance accounted for? |
|  | 4.9. Do predictors /feature selection and their assigned weights in the final model correspond to the results from the reported multivariable analysis  or ML ? |

*Red color indicate adjustment made for machine learning models

**Table S3.** Risk-of-bias assessment of each study by PROBAST questions.

| **Study** | 1.1 | 1.2 | 2.1 | 2.2 | 2.3 | 3.1 | 3.2 | 3.3 | 3.4 | 3.5 | 3.6 | 4.1 | 4.2 | 4.3 | 4.4 | 4.5 | 4.6 | 4.7 | 4.8 | 4.9 | **Overall risk** |
| --- | --- | --- | --- | --- | --- | --- | --- | --- | --- | --- | --- | --- | --- | --- | --- | --- | --- | --- | --- | --- | --- |
| Chen (2018)^14^ | PY | PY | Y | PY | PY | Y | PY | Y | PY | PY | NI | PY | NI | PY | NI | Y | Y | PY | Y | NI | ± |
| Hayashi (2021)^15^ | PY | Y | Y | NI | PY | Y | Y | Y | Y | PY | PY | PY | Y | PY | Y | Y | Y | PY | Y | Y | - |
| Huo (2021)^16^ | PY | Y | Y | NI | PY | PY | PY | Y | Y | PY | PY | PN | PY | NI | PY | Y | PY | PY | Y | Y | + |
| Sung (2021)^17^ | Y | Y | Y | NI | Y | Y | PY | Y | Y | PY | PY | PY | NI | NI | PY | Y | PY | PN | Y | Y | + |
| Tarkanyi (2022)^18^ | Y | Y | Y | Y | PY | Y | Y | Y | Y | Y | PY | PY | PY | PY | Y | Y | Y | PY | Y | Y | - |
| Thomas (2021)^19^ | Y | Y | Y | NI | PY | Y | Y | Y | Y | Y | PY | PN | PY | PY | Y | PY | Y | PY | Y | Y | + |
| Uchida (2021)^20^ | Y | PY | Y | PY | PY | Y | Y | Y | Y | NI | PY | PY | PN | PY | PN | Y | Y | PY | Y | Y | + |
| Wang (2022)^21^ | PY | PY | Y | PY | PY | Y | Y | Y | Y | PY | PY | Y | PY | PY | NI | PY | NI | PY | Y | PY | ± |

Abbreviations: **N**, not for low risk of bias; **n.a.**, not assessed; **NI**, no information for risk of bias assessment; **PN**, probably not for low risk of bias; **PY**, probably yes for low risk of bias; **Y**, yes for low risk of bias; **+**, indicated high risk of bias; **-**, indicated low risk of bias; **±**, indicated unclear risk of bias.

| **Table S4.** TRIPOD checklist for reporting quality assessment | |
| --- | --- |
| **Title & Abstract** | 1. Identify the study was development/validation/both, outcome of interest and mention and mention prehospital or machine learning |
|  | 2. Provide a summary of objectives, study design, setting, participants, sample size, predictors, outcome, statistical analysis, results, and conclusions. |
| **Background &Objectives** | 3a. Explain the medical context (including whether diagnostic or prognostic) and rationale for developing or validating the machine/deep model, including references to existing models. |
|  | 3b. Specify the objectives, including whether the study describes the development or validation of the model or both. |
| **Methods** | 4a. Describe the study design or source of data (e.g., randomized trial, cohort, or registry data), separately for the development and validation data sets, if applicable. |
|  | 4b. Specify the key study dates, including start of accrual; end of accrual; and, if applicable, end of follow-up. |
|  | 5a. Specify key elements of the study setting (e.g., primary care, secondary care, general population) including number and location of centres. |
|  | 5b. Describe eligibility criteria for participants. |
|  | 5c. Give details of treatments received, if relevant. |
|  | 6a. Clearly define the outcome that is predicted by the prediction model, including how and when assessed. |
|  | 6b. Whether any reporting on any actions to blind assessment of the outcome to other clinical data to be predicted. |
|  | 7a. Clearly define all predictors used in developing or validating the multivariable prediction model, including how and when they were measured |
|  | 7b. Report any actions to blind assessment of predictors for the outcome and other predictors |
|  | 8. Explain how the study size was arrived at. |
|  | 9. Describe how missing data were handled (e.g., complete-case analysis, single imputation, multiple imputation) with details of any imputation method. |
|  | 10a. Describe how predictors were handled in the analyses. |
|  | 10b. Specify type of model, all model-building procedures (including any predictor selection, hyperparameter selection if needed), and method for internal validation |
|  | 10c. For validation, describe how the predictions were calculated. |
|  | 10d. Specify all measures used to assess model performance and, if relevant, to compare multiple models. |
|  | 10e. Describe any model updating (e.g., recalibration) arising from the validation, if done. |
|  | 11. Provide details on how risk groups were created, if done. |
|  | 12. For validation, identify any differences from the development data in setting, eligibility criteria, outcome, and predictors. |

| **Table S4.** TRIPOD checklist for reporting quality assessment (continues) | |
| --- | --- |
| **Results** | 13a. Describe the flow of participants through the study, including the number of participants with and without the outcome and, if  applicable, a summary of the follow-up time. A diagram may be helpful. |
|  | 13b. Describe the characteristics of the participants (basic demographics, clinical features, available predictors), including the number  of participants with missing data for predictors and outcome. |
|  | 13c. For validation, show a comparison with the development data of the distribution of important variables (demographics, predictors  and outcome). |
|  | 14a. Specify the number of participants and outcome events in each analysis. |
|  | 14b. If done, report the unadjusted association between each candidate predictor and outcome. |
|  | 15a. Present the full prediction model to allow predictions for individuals (i.e., all regression coefficients, and model intercept or baseline survival at a given time point) or (i.e. links to the final model online (coding of predictors, code and final  parameters/coefficients, and with the architecture described in full in the article)). |
|  | 15b. Explain how to use the prediction model |
|  | 16. Report performance measures (with CIs, p-value or range) for the prediction model. |
|  | 17. If done, report the results from any model updating (i.e., model specification, model performance). |
| **Discussion** | 18. Discuss any limitations of the study (such as nonrepresentative sample, few events per predictor, missing data) |
|  | 19a. For validation, discuss the results with reference to performance in the development data, and any other validation data. |
|  | 19b. Give an overall interpretation of the results, considering objectives, limitations, results from similar studies, and other relevant evidence. |
|  | 20. Discuss the potential clinical use of the model and implications for future research. |
| **Other information** | 21. Provide information about the availability of supplementary resources, such as study protocol, Web calculator, and data sets. |
|  | 22. Give the source of funding and the role of the funders for the present study. |

*Red color indicate adjustment made for machine learning models

**Table S5.** Quality assessment of each study by TRIPOD checklist

| Study | 1 (D;V) | 2 (D;V) | 3a (D;V) | 3b (D;V) | 4a (D;V) | 4b (D;V) | 5a (D;V) | 5b (D;V) | 5c (D;V) | 6a (D;V) | 6b (D;V) | 7a (D;V) | 7b (D;V) | 8 (D;V) | 9 (D;V) | 10 a (D) | 10b (D) | 10c (V) | 10d (D;V) | 10e (V) | 11 (D;V) | 12 (V) | 13a (D;V) | 13b (D;V) | 13c (V) | 14a (D) | 14b (D) | 15a (D) | 15b (D) | 16 (D;V) | 17 (V) | 18 (D;V) | 19a (V) | 19b (D;V) | 20 (D;V) | 21 (D;V) | 22 (D;V) |
| --- | --- | --- | --- | --- | --- | --- | --- | --- | --- | --- | --- | --- | --- | --- | --- | --- | --- | --- | --- | --- | --- | --- | --- | --- | --- | --- | --- | --- | --- | --- | --- | --- | --- | --- | --- | --- | --- |
| Chen (2018)^14^ | Y | Y | Y | Y | Y | Y | N | PY | Y | Y | PY | Y | N | Y | N | N | Y | na | Y | na | na | na | Y | N | na | Y | na | N | Y | Y | na | Y | na | Y | Y | N | Y |
| Hayashi  (2021)^15^ | Y | Y | Y | Y | Y | Y | Y | Y | na | Y | PY | Y | Y | Y | Y | Y | Y | na | Y | na | na | na | Y | PY | na | Y | na | PY | PY | Y | na | Y | na | Y | Y | Y | Y |
| Huo  (2021)^16^ | Y | Y | Y | Y | Y | Y | Y | Y | na | Y | PY | Y | Y | na | Y | Y | Y | na | Y | na | Y | na | Y | PN | na | PY | na | PY | PY | Y | na | Y | na | Y | Y | N | Y |
| Sung  (2021)^17^ | PY | Y | Y | Y | Y | PY | PY | Y | na | Y | Y | Y | Y | Y | Y | Y | Y | na | Y | na | na | na | Y | PY | na | PN | na | PY | PY | PN | na | Y | na | Y | Y | Y | Y |
| Tarkanyi  (2022)^18^ | PY | Y | Y | Y | Y | PY | PY | Y | na | Y | Y | Y | Y | Y | Y | Y | Y | na | Y | na | na | na | Y | PY | na | PN | na | PY | PY | PN | na | Y | na | Y | Y | Y | Y |
| Thomas  (2021)^19^ | Y | Y | Y | Y | Y | Y | Y | Y | na | PY | na | Y | na | Y | Y | Y | PY | na | Y | na | Y | na | Y | Y | na | Y | na | PY | PY | PN | na | Y | na | Y | Y | Y | Y |
| Uchida  (2021)^20^ | Y | Y | Y | Y | Y | Y | Y | Y | na | Y | na | Y | Y | Y | PY | Y | PY | na | Y | na | na | na | Y | Y | na | Y | na | PY | PY | Y | na | Y | na | Y | Y | Y | Y |
| Wang  (2022)^21^ | Y | Y | Y | Y | Y | Y | NI | Y | na | Y | Y | Y | Y | Y | NI | Y | PY | na | Y | na | na | na | Y | Y | Y | Y | na | na | PY | Y | na | Y | na | Y | Y | PY | Y |
| **D**, items relevant to the development of a prediction model; **D;V**, items relevant to both of the development and validation of a prediction model; **N**, didn’t report; **na**, not assessed; **PN**, probably no; **PY**, probably yes; **V**, items relevant to the validation of a prediction model; **Y**, reported. | | | | | | | | | | | | | | | | | | | | | | | | | | | | | | | | | | | | | |


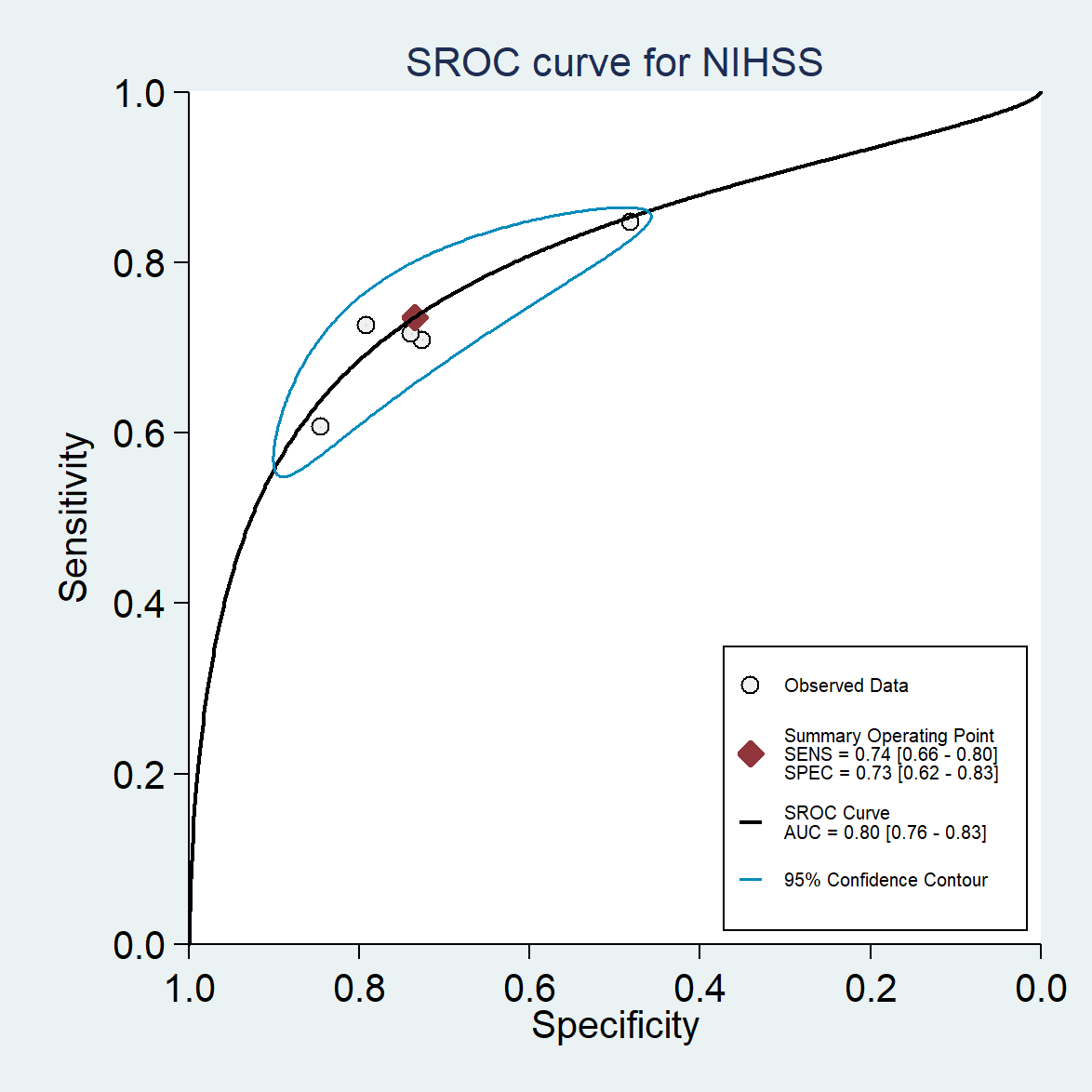


**Figure S1A.** Summary ROC curve (SROC) of National Institute of Health stroke scale (NIHSS)


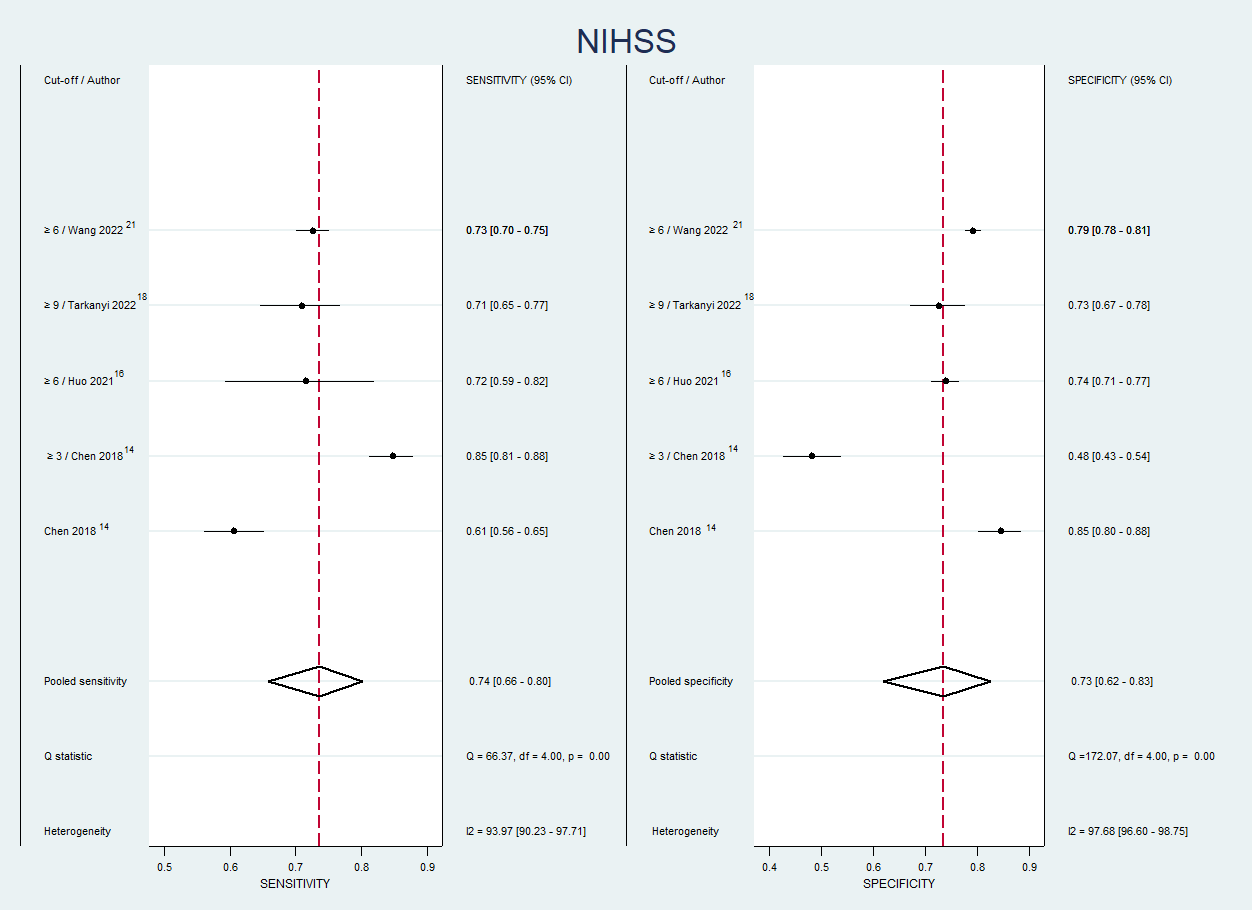


**Figure S1B.** A forest plot demonstrating the individual and pooled sensitivity and specificity of National Institute of Health stroke scale (NIHSS).


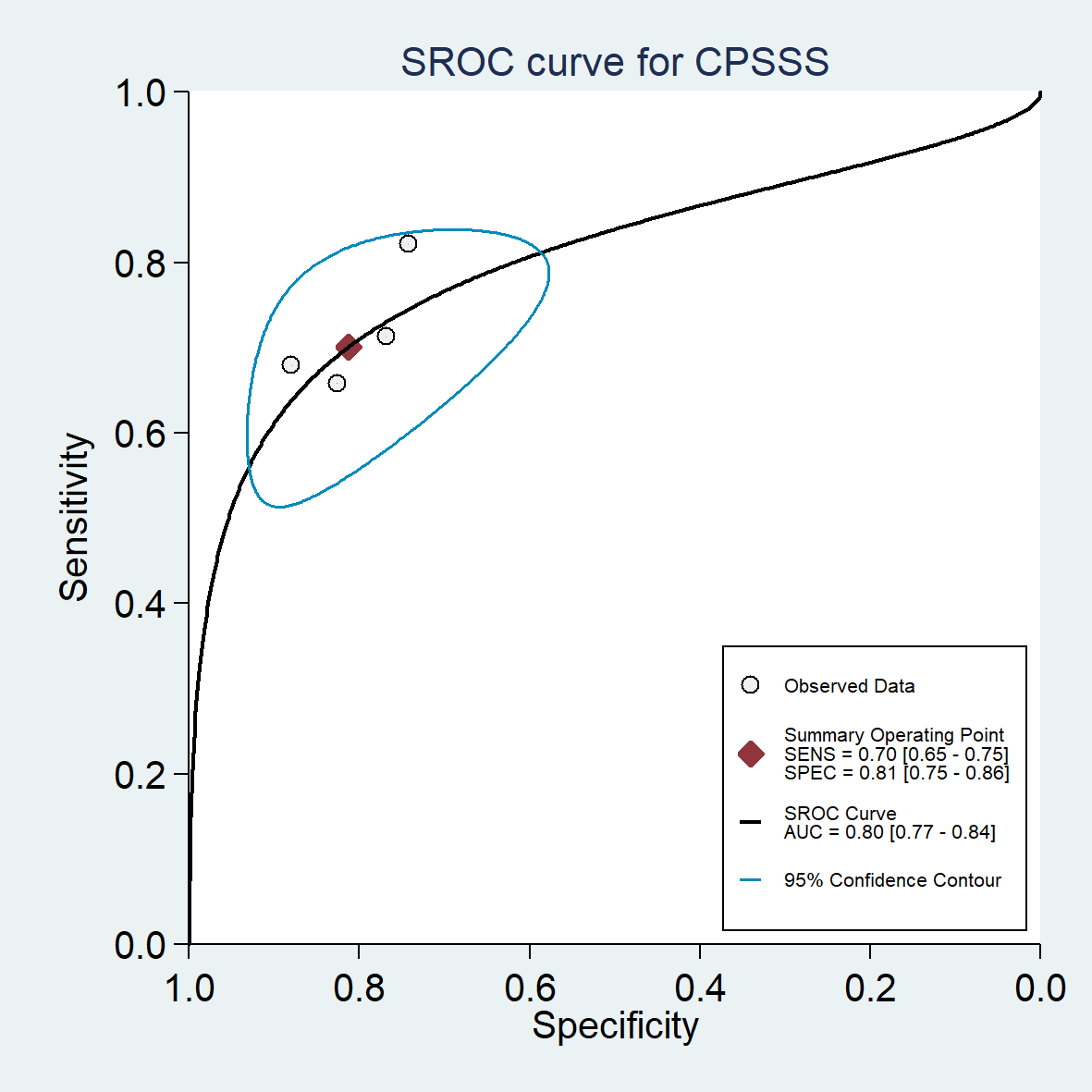


**Figure S2A.** Summary ROC curve (SROC) of Cincinnati Prehospital Stroke Severity Scale (CPSSS).


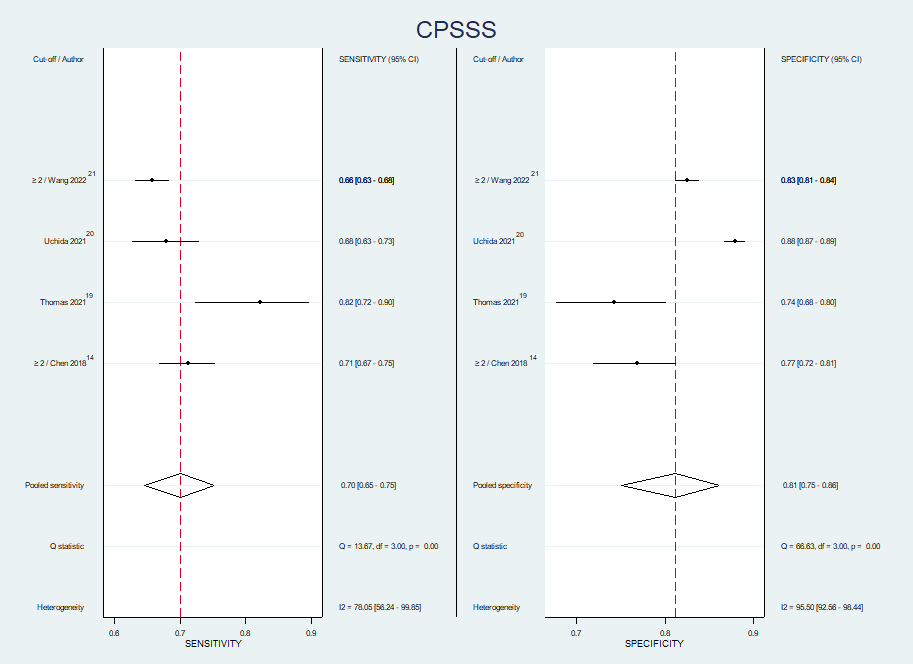


**Figure S2B.** A forest plot demonstrating the individual and pooled sensitivity and specificity of Cincinnati Prehospital Stroke Severity Scale (CPSSS).


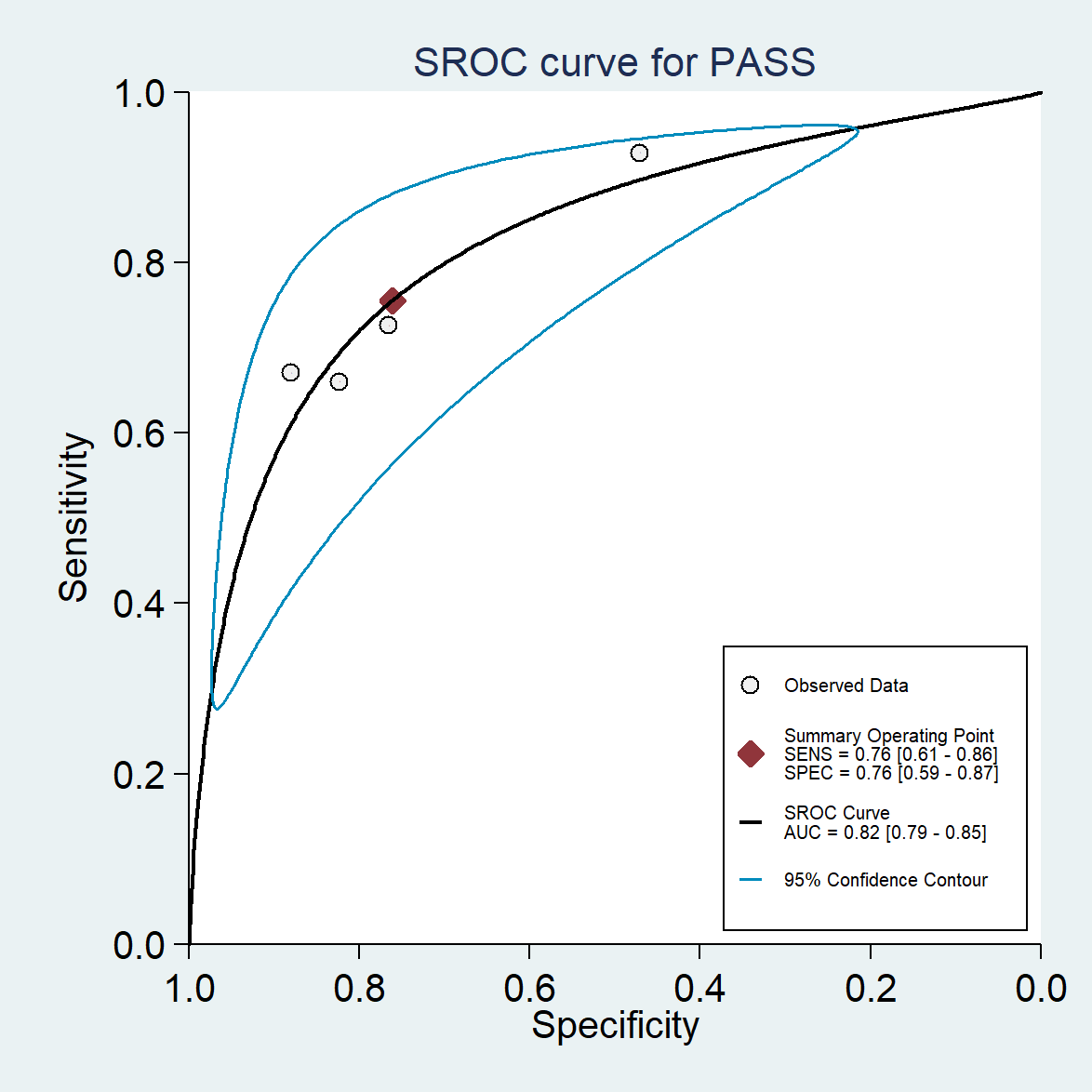


**Figure S3A.** Summary ROC curve (SROC) of Pre-hospital Acute Stroke Severity (PASS).


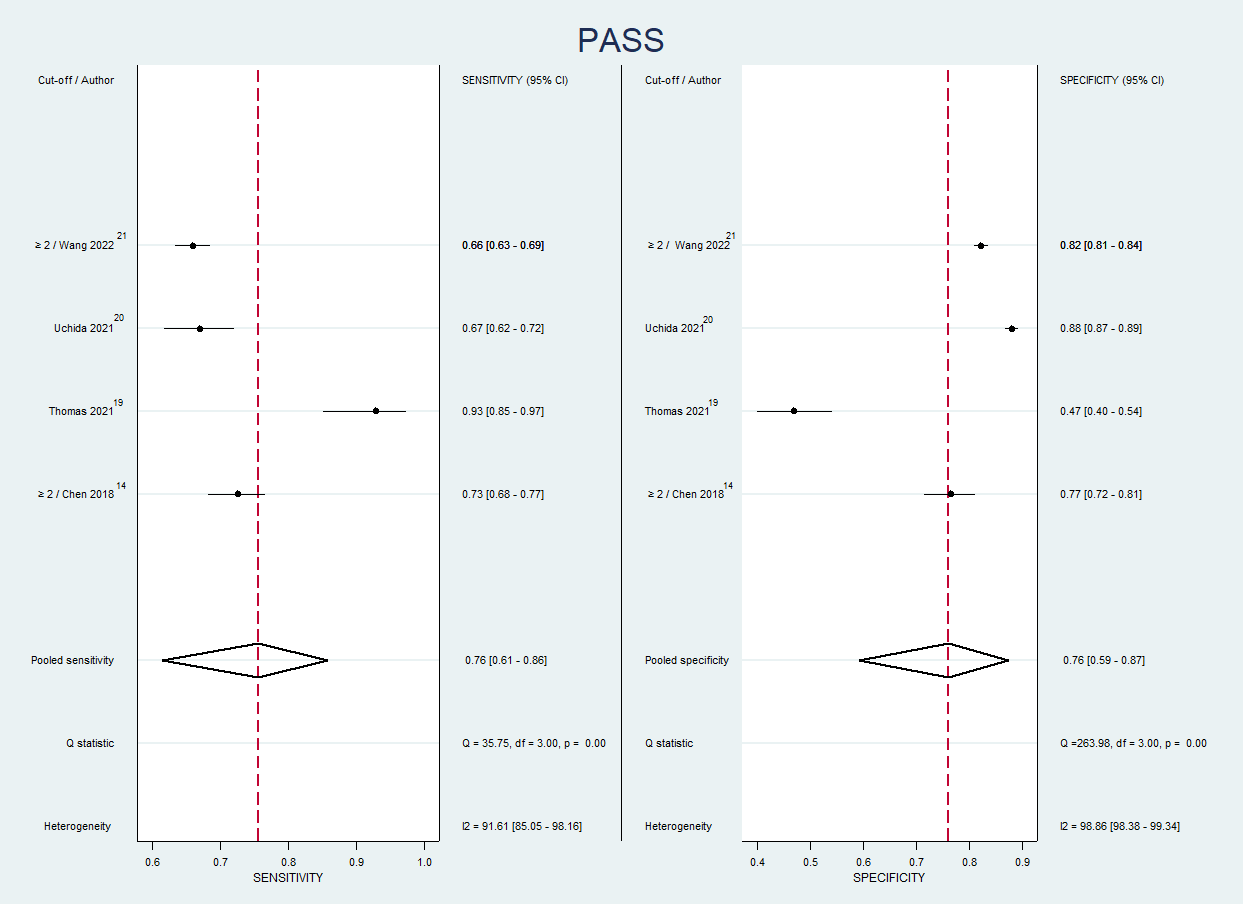


**Figure S3B.** A forest plot demonstrating the individual and pooled sensitivity and specificity of Pre-hospital Acute Stroke Severity (PASS).


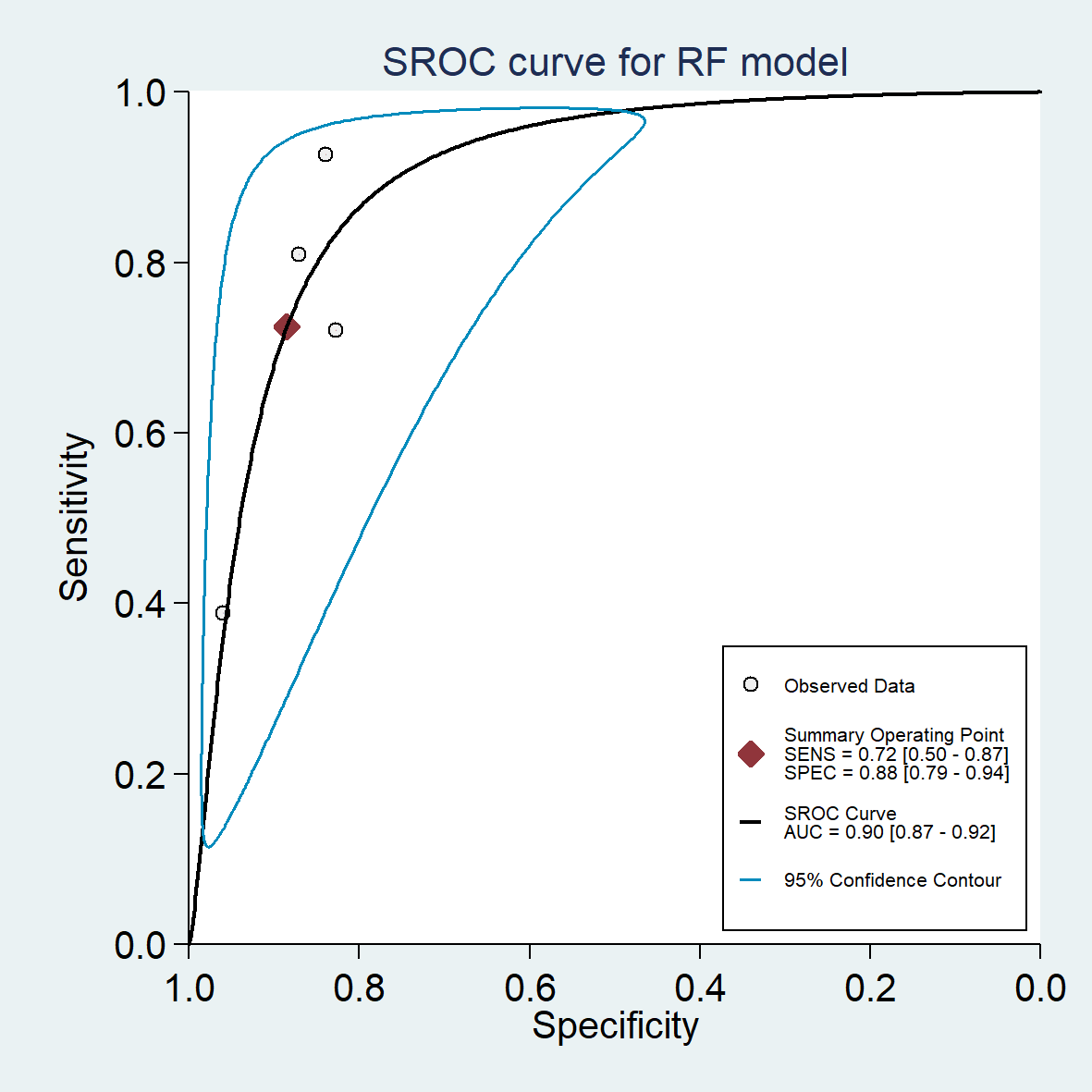


**Figure S4A.** Summary ROC curve (SROC) of Random forest (RF) model.


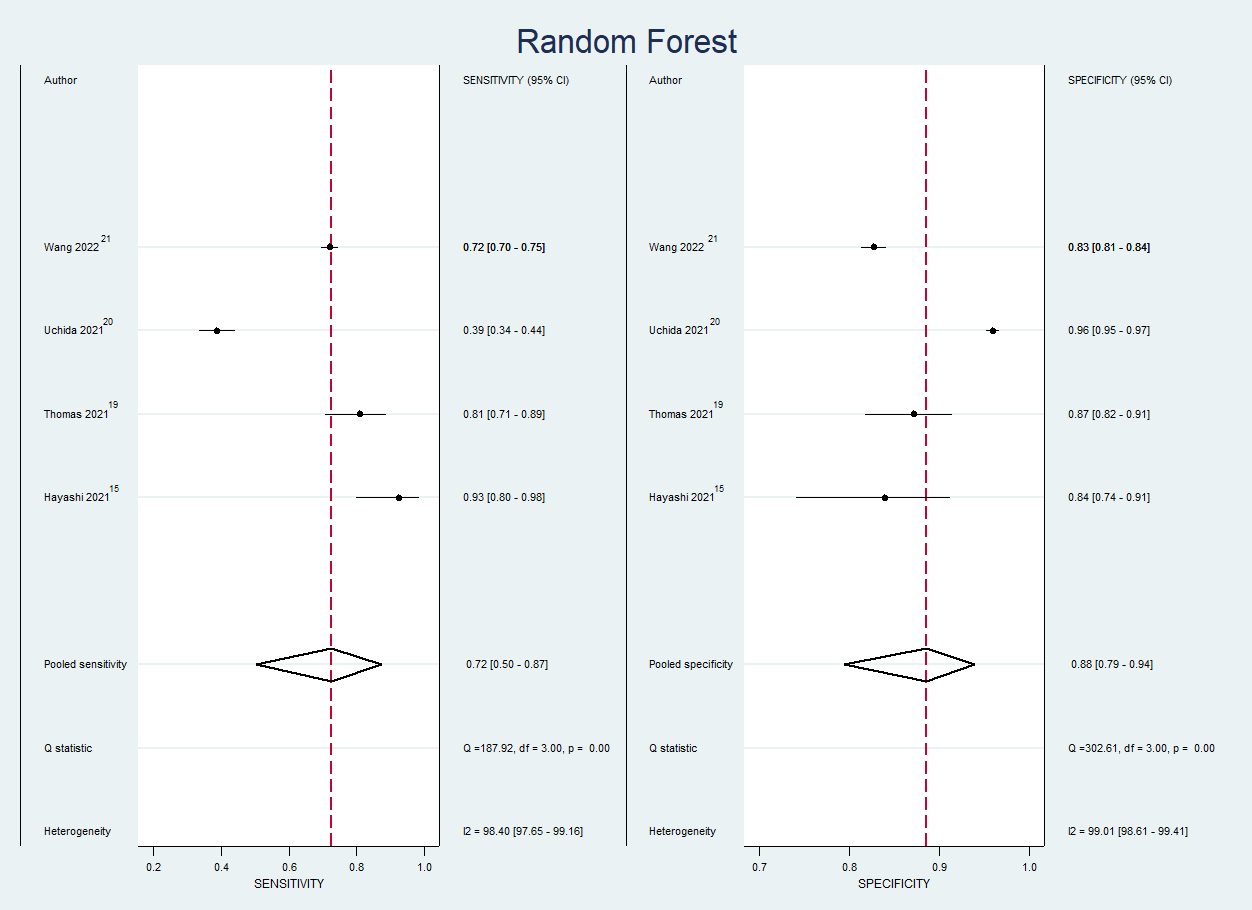


**Figure S4B.** A forest plot demonstrating the individual and pooled sensitivity and specificity of Random forest (RF) model.


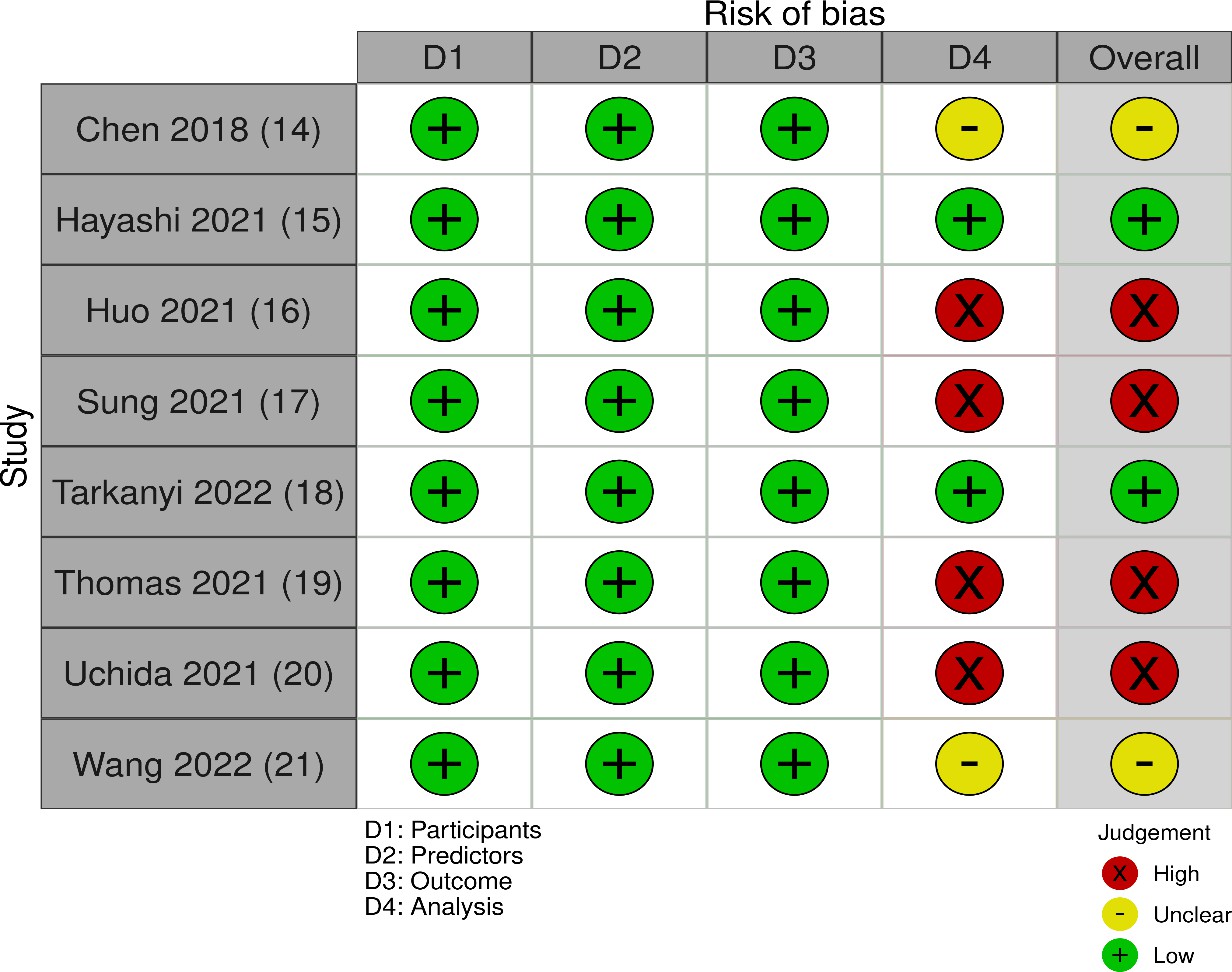


**Figure S5.** Risk-of-bias assessment of each study by PROBAST domains.^30^


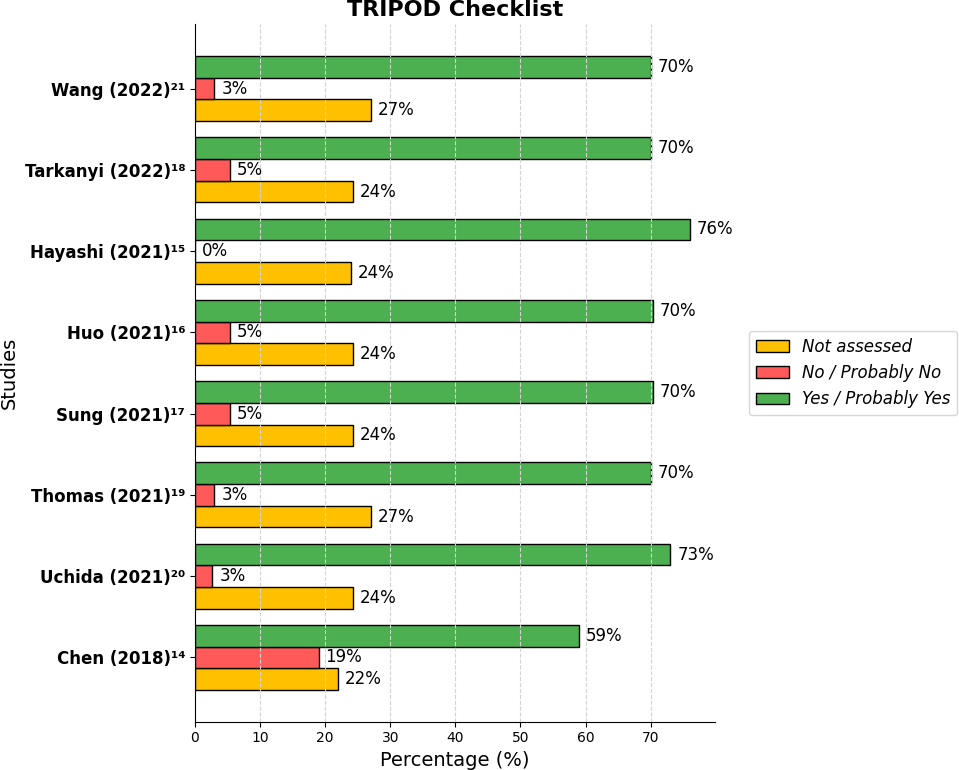


**Figure S6.** Bar chart depicting the percentage of quality reporting in the included studies using TRIPOD checklist.
